# Considerations for multimodal prehabilitation in women with gynaecological cancers: a scoping review using realist principles

**DOI:** 10.1186/s12905-022-01882-z

**Published:** 2022-07-19

**Authors:** Rhia Kaur Saggu, Phillip Barlow, John Butler, Sadaf Ghaem-Maghami, Cathy Hughes, Pernilla Lagergren, Alison H. McGregor, Clare Shaw, Mary Wells

**Affiliations:** 1 Department of Nutrition and Dietetics, Imperial College Healthcare NHS Trust, Charing Cross Hospital, 13th Floor Laboratory Block, Fulham Palace Road, London, W6 8RF UK; 2 Medical Library, Chelsea and Westminster Campus, Imperial College London, Fulham Road, London, UK; 3 Gynaecological Unit, The Royal Marsden Hospital, Fulham Road, London, UK; 4 Department of Surgery and Cancer, Imperial College London, Hammersmith Campus, Du Cane Road, London, UK; 5 Department of Surgery and Cancer, Imperial College Healthcare NHS Trust, University of Surrey, Guildford, UK; 6 Department of Surgery and Cancer, Imperial College London, London, UK; 7 Department of Molecular Medicine and Surgery, Karolinska Institutet, Stockholm, Sweden; 8 Musculoskeletal Lab, Department of Surgery and Cancer, Imperial College London, London, UK; 9 Royal Marsden and Institute of Cancer Research Biomedical Research Centre, London and Sutton, UK; 10 Department of Surgery and Oncology, Nursing Directorate, Imperial College Healthcare NHS Trust, Charing Cross Hospital, Imperial College London, Fulham Palace Road, London, UK

**Keywords:** Prehabilitation, Gynaecology, Cancer, Pre-operative care

## Abstract

**Background:**

There is increasing recognition that prehabilitation is important as a means of preparing patients physically and psychologically for cancer treatment. However, little is understood about the role and optimal nature of prehabilitation for gynaecological cancer patients, who usually face extensive and life-changing surgery in addition to other treatments that impact significantly on physiological and psychosexual wellbeing.

**Review question:**

This scoping review was conducted to collate the research evidence on multimodal prehabilitation in gynaecological cancers and the related barriers and facilitators to engagement and delivery that should be considered when designing a prehabilitation intervention for this group of women.

**Methods:**

Seven medical databases and four grey literature repositories were searched from database inception to September 2021. All articles, reporting on multimodal prehabilitation in gynaecological cancers were included in the final review, whether qualitative, quantitative or mixed-methods. Qualitative studies on unimodal interventions were also included, as these were thought to be more likely to include information about barriers and facilitators which could also be relevant to multimodal interventions. A realist framework of context, mechanism and outcome was used to assist interpretation of findings.

**Results:**

In total, 24 studies were included in the final review. The studies included the following tumour groups: ovarian only (n = 12), endometrial only (n = 1), mixed ovarian, endometrial, vulvar (n = 5) and non-specific gynaecological tumours (n = 6). There was considerable variation across studies in terms of screening for prehabilitation, delivery of prehabilitation and outcome measures. Key mechanisms and contexts influencing engagement with prehabilitation can be summarised as: (1) The role of healthcare professionals and organisations (2) Patients’ perceptions of acceptability (3) Factors influencing patient motivation (4) Prehabilitation as a priority (5) Access to prehabilitation.

**Implications for practice:**

A standardised and well evidenced prehabilitation programme for women with gynaecological cancer does not yet exist. Healthcare organisations and researchers should take into account the enablers and barriers to effective engagement by healthcare professionals and by patients, when designing and evaluating prehabilitation for gynaecological cancer patients.

**Supplementary Information:**

The online version contains supplementary material available at 10.1186/s12905-022-01882-z.

## Introduction

Prehabilitation offers the opportunity to improve patients’ physical and mental function, through buffering the deconditioning related to cancer treatments between the time of diagnosis and recovery [1]. Prehabilitation has been shown to reduce pulmonary and overall morbidity and improve post-operative gait, cardiovascular function and urinary continence in those undergoing major cancer surgeries [1, 2] It also has the potential to improve health related quality of life in the longer term [3]. Multimodal programmes generally consist of a combination of medical management, physical activity, nutrition and psychological wellbeing and are considered more effective than standard care approaches or unimodal interventions [4, 5]. Gynaecological cancers consist of vulvar, cervical, vaginal, endometrial, and ovarian tumours. The latter in particular are associated with increased mortality and morbidity, often due to late and advanced presentation [6, 7]. Women with endometrial cancer have better survival overall, but over 50% are obese and therefore at risk of cardiovascular disease and other co-morbidities [8]. Suboptimal conditioning prior to surgery is likely to exacerbate post-treatment side-effects already experienced by gynaecological cancer patients undergoing chemotherapy and radiotherapy, such as gastrointestinal and sexual dysfunction, urinary incontinence, menopause and lymphoedema [9, 10]. This in turn, costs healthcare services a significant amount of money in rehabilitation [11].

The potential for prehabilitation in gynaecological cancers has been recognised [12] but little is known about the specific prehabilitation needs of women facing gynaecological cancer treatment and the barriers and facilitators influencing engagement in and outcomes of prehabilitation. This is important to the targeting and personalisation of prehabilitation programmes to enhance uptake and effectiveness [13].

## Methodology

The aim of this scoping review was to explore the empirical and theoretical evidence for multimodal prehabilitation amongst women with gynaecological cancers, with particular emphasis on the enablers and barriers to prehabilitation delivery, engagement, and adherence in this patient group. Scoping reviews are particularly relevant for examining the extent, range and nature of the evidence on a topic and for summarising findings from a heterogeneous body of knowledge [14]. This review used a realist lens to enable a detailed exploration of factors likely to influence the success of a complex intervention, such as prehabilitation [15]. Realist approaches focus on the contexts and mechanisms that lead to particular outcomes, thus helping to explain how and why interventions may or may not work [16]. Other reviewers have combined scoping and realist approaches to understand complex contexts [17].

This review follows the Joanna Briggs Institute (JBI) guidelines for scoping reviews [18], Preferred Reporting Items for Systematic reviews and Meta-Analyses extension for Scoping Reviews (PRISMA-ScR) checklist [14]. The review protocol has been published in an open access forum [19].

The review was conducted following the key steps outlined by JBI:

1. Define the review questions 2. Determine the inclusion criteria 3. Search strategy 4. Evidence screening and selection 5. Data extraction 6. Data analysis 7. Presentation of the results [18].

*Step* 1 *Define the review questions* Given the complexity of prehabilitation as an intervention, it is important not only to understand what has worked or is perceived to work based on measured or predicted outcomes, but also the mechanisms and the context which may operate as facilitators and barriers, and thus influence the success of a prehabilitation intervention [20]. Our research questions were:

1. How does the gynaecological cancer literature define ‘prehabilitation’?
2. What are the intended and unintended outcomes for gynaecological cancer patients participating in a prehabilitation programme?
3. What are the key components, skills and contexts required by the healthcare team to implement a successful prehabilitation programme in this population?
4. What are the facilitators and barriers to engaging in prehabilitation amongst patients with gynaecological cancers?

*Step* 2 *Determine the inclusion criteria* To be included in this review, all studies needed to investigate and or report on the role, impact and/or influencers surrounding prehabilitation, from the perspectives of either gynaecological cancer patients and/or health professionals. All study designs were included in this scoping review, on the basis that that they met the inclusion criteria outlined in Table 1. Study abstracts as well as protocols for ongoing trials of relevant multimodal prehabilitation interventions were included in the final review as the authors felt these provided key insights into the nature, delivery and intended outcomes of prehabilitation interventions. Quantitative studies and protocols were included only if they addressed multimodal prehabilitation programmes. Qualitative studies describing unimodal programmes were also included, as their results were likely to be beneficial in understanding facilitators and barriers which could also be relevant to taking part in multimodal programmes.

**Table 1:** Eligibility criteria

Inclusion criteria	
Population	Clinicians e.g., doctors, nurses and allied health professions involved in managing gynaecological cancers Adult female patients diagnosed with a gynaecological malignancy i.e., cervical, vulvar, vaginal, endometrial, or ovarian Caregivers and/or relatives of patients described above
Intervention	Multimodal* interventions prior to surgery in isolation or in combination with an ERAS** intervention Qualitative studies exploring views and opinions of prehabilitation (uni/multimodal) amongst gynaecological cancer patients
Comparator	Any intervention or usual care (within a randomised trial)
Outcomes	The facilitators and enablers to engagement in prehabilitation The barriers to engagement and adherence with prehabilitation The intended and unintended outcomes of participating in prehabilitation The effectiveness of prehabilitation programmes
Healthcare context	Any setting that provides care to adult cancer populations e.g., hospital, ambulatory care, outpatient/ community care, primary care, digital platforms
Study design	Qualitative, quantitative, or mixed methods studies. If relevant existing systematic reviews are identified, their primary papers will be included. Published up to and including September 2021 Protocols for ongoing trials of relevant prehabilitation interventions which meet the inclusion criteria
*Exclusion criteria*	
Population	Studies addressing tumour groups or sites other than those listed above in the inclusion criteria
Intervention	Interventions focussing on single pre-operative interventions which are not part of a multimodal approach
Study design	Social media posts, podcasts and blogs will be excluded
Language	Articles published in a language other than English. Translation from other languages will not be possible due to lack of resources

*Multimodal = A programme delivering two or more non-pharmacological interventions e.g., nutrition and physical activity or psychological counselling, structured exercise and psychological wellbeing. **ERAS = Enhanced recovery after surgery

Any articles published in a language other than English, were excluded due to limited translation resources.

*Step* 3 *Search Strategy* All searches for relevant literature were carried out by the research librarian following discussion with the research team to predefine search terms (see Additional file 1). Articles were retrieved on the 6th October 2021 using the major search terms ‘gynaecology’ ‘cancer’ and ‘prehabilitation’ from the date of database inception to September 2021. A comprehensive set of seven databases were searched using the National Health Service’s Healthcare Database (HDAS) to encompass medical, nursing, allied health and psychological literature relevant to multimodal prehabilitation. These included Allied and Complementary Medicine Database (AMED), British Nursing Index (BNI), Cumulative Index to Nursing and Allied Health Literature (CINAHL), Embase, Emcare, Medical Literature Analysis and Retrieval System Online (MEDLINE) and Psychological Information Database (PsycINFO). Since the search was conducted, HDAS has been discontinued, however, the underlying databases remain available via other platforms and the same search strategy can be replicated. The search was also conducted in the Cochrane Library platform, across the Cochrane Database of Systematic Reviews, Central Register of Clinical Trials and Cochrane Clinical Answers, using an identical set of keywords and subject headings to the MEDLINE version of the original search. Additionally a search for grey literature was conducted in National Institute for Health and Care Excellence (NICE) Evidence Search and Turning Research into Practice (TRIP) Database, and using the search engine Google. In depth search strategies can be found in Additional file 1: Tables S1 and S2. For completeness, the reference lists of all included papers were reviewed for possible inclusion.

*Step* 4 *Evidence Screening and Selection Duplications* were removed from all retrieved articles using HDAS’ deduplication function. All retrieved abstracts were uploaded to Covidence, for independent screening by the first and last author. Full texts of papers for possible inclusion were then reviewed by the same authors using Covidence [21]. Any disagreement in decision making was discussed and consensus was reached between the two reviewers at each stage of the screening, thus, a third reviewer was not required. We did not conduct critical appraisal as this is not generally recommended in scoping reviews [18].

*Steps* 5 and 6 *Data extraction and analysis* All data were extracted using the JBI Reviewers’ Manual as a guide [22]. A summary table was compiled to include details of title, year of publication, country, study design, sample, key findings related to the scoping review, strengths and limitations (Table 2). Additionally, data on all interventions, including those described within registered trial protocols, were categorised using the Template for Intervention Description and Replication (TIDieR) checklist [23] (Table 3). This allows a more detailed understanding of the components of interventions, how they are delivered and tailored and how they are evaluated. Findings related to barriers and facilitators to engagement with prehabilitation were considered in relation to the Context-Mechanism-Outcome framework [22].

**Table 2:** Summary of all studies qualitative and quantitative studies meeting eligibility criteria and subsequently used in the present scoping review

Title and year	Country of origin	Aims/purpose	Study population/sample size	Study design/type	Key findings related to the scoping review objectives	Strengths/limitations
SOPHIE Trial: Surgery in Ovarian Cancer with PreHabilitation in ERAS* 2021–2024 [23]	Spain	To determine the efficacy of multimodal prehabilitation in decreasing postoperative complications in patients undergoing gynaecological cancer surgery of high complexity	N = 146 advanced ovarian cancer patients N = 73 in each arm	Randomised controlled trial (PROTOCOL)	Planned outcomes: Aerobic activity, physical activity, post-operative complication, length of stay and associated costs	Strengths: large sample size Cost-effectiveness analysis will be undertaken as part of outcomes Limitations: patients excluded if unable to undertake a minimum of 3 weeks prehabilitation prior to surgery. Not highly translatable due to different surgical pathways
Prehabilitation in patients with advanced stage ovarian cancer planned for interval debulking surgery (PHOCUS) 2020–2022 [24]	Prague	To comprehensively evaluate a trimodal prehabilitation pathway for patients with extensive ovarian cancer	N = 50 advanced ovarian cancer patients N = 25 per arm	Randomised controlled trial (PROTOCOL)	Planned outcome: change in 6MWT**	Strengths: trimodal prehabilitation programme (nutrition, physical activity, and psychology) Limitations: no detailed description of individual components Relatively small sample size (25 per arm) Those undergoing primary debulking surgery are excluded
Home-based telemonitoring program for functional recovery and symptoms in gastrointestinal, genitourinary or gynecologic cancer patients undergoing abdominal surgery 2021- 2024 [25]	USA	To compare a home-based telemonitoring multimodal prehabilitation programme to standard surgeon only care in improving recovery and stopping complications within 30 days after surgery in patients scheduled for abdominal surgery	N = 332 cancer patients of various tumour groups and disease stages	Randomised controlled trial (PROTOCOL)	Planned outcomes: change in daily step count and post-operative complications. Qualitative study on the prehab programme. Change in sedentary time, sleep and general symptoms	Strengths: study includes multiple tumour groups within gynaecology Entirely remote prehabilitation programme with the opportunity to connect with the treating team face to face if required Limitations: patients only included if they were able to read/understand English and Spanish
Prehabilitation plus ERAS vs ERAS in gynaecological surgery 2020–2022 [26]	Brazil	To test the effectiveness of a trimodal prehabilitation programme in addition to ERAS compared with ERAS alone in patients undergoing gynaecologic surgery for diagnosed or suspicious gynaecologic malignancies	N = 194 females diagnosed with or suspicion of gynaecologic malignancy	Single blinded (investigator) randomised controlled trial (PROTOCOL)	Planned outcomes: complications, readmissions, intensive care admissions, health related quality of life, compliance to ERAS protocol, changes in anxiety/depression, changes in functional capacity, changes in muscle strength, change in body mass, hospital stay	Strengths: The consultants are blinded to the intervention, but the allied health professionals are not Prehabilitation intervention is as short as 2–3 weeks which is more translatable to patients with short durations between diagnosis and surgery Includes participants with a suspicion of gynaecologic cancer Not limited to ovarian cancer patients
Connected Prehabilitation program during neo adjuvant chemotherapy 2022–2027 [27]	France	To investigate whether carrying out a connected supervised home based, tailored programme (using activity watches, scales, and a phone application) during NACT*** improves physical fitness and positively improve post-operative outcomes	N = 136 ovarian cancer patients due to undergo NACT	Randomised controlled trial (PROTOCOL)	Planned outcomes: primary outcome: VO_2_ max^$^ comparison between arms Secondary outcomes: muscular strength, Hospital depression and anxiety score, cancer related quality of life, nutritional outcomes	Strengths: Entirely remote prehabilitation programme which is tailored to individual requirements based on activity tracking and smart scales Limitations: participants without access to a computer or smartphone will be excluded
Gyn Onc Prehab Study 2020–2022 [28]	USA	To examine the impact of a trimodal prehabilitation programme with a unimodal programme (physical activity only)	N = 164 ovarian, endometrial and cervical cancer patients	Randomised controlled trial (PROTOCOL)	Planned outcomes: Primary outcomes: Change in 6MWT and grip strength Secondary outcomes: readmission, complication, patient satisfaction, quality of life, treatment completion	Strengths: first trial within gynaecological cancers to compare trimodal prehabilitation with unimodal prehabilitation Limitations: non-English speaking participants are excluded as well as those with a poor performance status. Not representative of gynaecological cancer patients requiring surgery
PROADAPT- ovary/ EWOC-2 2020–2023 [29]	France	To determine impact of multimodal prehabilitation in patients over 70 years of age	N = 292 advanced ovarian cancer patients over the age of 70 or over 60 years if they have a significant comorbidity	Randomised controlled trial (PROTOCOL)	Planned outcomes: post treatment complication, health related quality of life. Progression free survival over 2 years. Improved 6MWT. Improvements in SF-36^ and overall survival [2 years]	Strengths: based on the logic change model: the rehabilitation model which has been validated by an expert group Standardised geriatric intervention which is being co-constructed on a multi-professional and multi-disciplinary basis that encompasses the period before surgery, immediately after surgery and discharge
Training-Ovary 01 multicenter randomized study comparing neoadjuvant chemotherapy for patients managed for ovarian cancer with or without a connected prehabilitation programme 2021–2024 [30]	France	To trial whether a connected prehabilitation programme during NACT will improve physical capacity prior to surgery for advanced ovarian cancer patients	136 patients with advanced ovarian cancer (stage iii-iv) undergoing NACT N = 66 per arm	Randomised controlled trial (PROTOCOL)	Planned outcomes: primary: to determine whether prehab improves physical conditioning prior to surgery compared with baseline. Outcome measure VO2max Secondary: nutritional status, physical fitness, psychological status	Strengths: Follow up period of 5 years Limitations: excludes those without computers and smartphones
F4S PREHAB Trial Multimodal intensive Prehabilitation in high impact surgery to reduce postoperative complications 2021–2023 [31]	Denmark	Understand the effects of prehabilitation on clinical outcomes, the underlying mechanism and cost efficiency of prehabilitation	Target N = 2380 Multiple tumour groups including ovarian, endometrial, and vulvar scheduled for high impact surgery	Stepped wedge cluster randomised controlled trial (PROTOCOL)	Planned outcomes: Primary outcome: Post-operative complications (Clavien-Dindo Score and Comprehensive complication Index) Secondary outcomes: Individual patient level: Length of stay (days), physical fitness (VO_2_ max, SQUASH^£^ questionnaire), nutritional status (body weight, fat free mass PG SGA-SF^#^), mental health (SF-36 questionnaire), intervention adherence Mechanistic level: Innate immune response Hospital efficiency level: Costs due to complications, costs due to length of stay, cost-effectiveness Macro-economic level: Changes in patient volumes and shifts in care between 2^nd^ and 1^st^ line healthcare	Strengths:large multicentre trial Multiple outcome measures Limitations: excluded people with an inability to read or understand Dutch No description of individual components of prehabilitation
Impact of a remote Prehabilitation programme in reducing delays to patients having surgery for advanced gynaecological cancer 2021 [32]	UK	To implement a remote prehabilitation programme to improve physical fitness, emotional wellbeing and reduce delays to surgery	N = 25 ovarian cancer patients undergoing 3–6 cycles of NACT prior to surgery Mean age- unknown	Cohort study (ABSTRACT)	Out of 25 patients who enrolled in the prehabilitation programme, 1 patient had surgery delayed due to lack of optimisation In a cohort of 25 people who did not receive prehabilitation, 6 people had a delay in having surgery	Strengths: both groups had similar demographic variables and treatment pathway as per authors Limitations: no sample data available to view Small patient group, single centre trial
A tertiary centre experience of prehabilitation for surgical ovarian cancer patients receiving neoadjuvant chemotherapy: The Royal Mile- Marsden Integrated Lifestyle and Exercise programme 2019 [33]	UK	To describe the initial experience of establishing a prehabilitation programme for ovarian cancer patients undergoing NACT at a London based tertiary cancer centre	N = 18 patients with advanced ovarian cancer receiving NACT Mean age- 73 years	Cohort study (ABSTRACT)	18/18 patients received at home exercise advice and nutrition advice from a nurse specialist 9/18 patients had low haemoglobin of which 6 needed intervention 5/18 patients were malnourished and referred for urgent dietetic review with oral nutritional supplementation 3/18 patients were selected to receive hospital-based exercise but all could not attend due to cancer related symptoms and other comorbidities. Another barrier was transport to the hospital Moving forward, the authors propose an entirely home-based exercise programme	Strengths: pilot (first in the centre) Trialled remote and face-face Limitations: small cohort, single centre No outcomes documented in terms of delays or post-op No data on demographics other than age
Prehab matters- a prehabilitation service for cancer patients undergoing major abdominal surgery 2019 [34]	UK	To report outcomes of a newly introduced prehabilitation service in Liverpool for patients undergoing major abdominal surgery	N = 1/32 gynaecological cancer patient	Cohort study (ABSTRACT)	Of the prehabilitation cohort, 12/32 suffered a complication post-surgery. Median length of stay in hospital was 6 days At 6 weeks follow up, BMI^↓^ was maintained, quality of life restored to baseline and 6MWT improved from 484 to 539 m Survey: 91% more able to cope with surgery 86% more like to make long term changes 60% said family likely to do the same	Strengths: prospective study so all data collected in real time Limitations: only 1 gynaecologic cancer patient so relevance of results poor Very poor retention. Of 142 patients who enrolled at baseline, only 33 patients attended post-operative follow up No data to compare outcomes from a cohort who did not receive prehabilitation
Prehabilitation to enhance post-operative recovery for an octogenarian following robotic-assisted hysterectomy with endometrial cancer 2012 [35]	Canada	To describe the impact of a multimodal prehabilitation programme on an 88 year old’s post-operative outcome	N = 1 endometrial cancer patient	Case study	Improvement in 6MWT and SF-36 at 4 and 8 weeks post surgery Self-reported improvement in concentration and mood Marginal improvement in dietary intake but protein and energy intake remained suboptimal	Strengths: one of the studies to highlight the benefit of prehabilitation for gynaecologic cancer patients Limitations: Case study based on the findings of one patient
Frequency of sarcopenia, sarcopenic obesity and changes in physical function in surgical oncology patients referred for prehabilitation 2021 [36]	USA	To describe the frequency of sarcopenia and sarcopenic obesity in a cohort of cancer patients referred for prehabilitation	N = 7/99 gynaecological cancer patients Mean age- 72 years	Cohort study	8/99 people did not have surgery due to poor performance status 9% underweight compared with 34% overweight and 27% obese 49% of patients were sarcopenic based on baseline CT scan, of which 28% fulfilled the criteria of being ‘sarcopenic obese’ Of this, 39% were sarcopenic with abnormal sit to stand and grip strength at baseline Baseline: Entire cohort had 6-min walk test, grip strength and × 5 sit to stand measures below normal for age and sex After following 30–90 days of prehabilitation, there was a significant improvement in above measures in both sarcopenic and non-sarcopenic individuals. The prehabilitation time duration did not significantly impact on distance covered in 6-min walk test Sarcopenia did not limit the potential of patients to improve functionally over the pre-operative period. Focus should be on lower limb training and grip strength as they could impact activities of daily living	Strengths: Study provided unique benefit of prehabilitation—improving function in both sarcopenic and non-sarcopenic patients Limitations: Study uses retrospective data No information on nutrition/weight history Evidence based definition of sarcopenia is required for future Difficult to derive direct impact on gynaecologic cancer patients Non-diverse ethnic sample (majority white)
Implementing prehabilitation as part of enhanced recovery after surgery (ERAS) efforts at a comprehensive cancer centre: A team-based approach 2018 [37]	USA	To utilise validated screening tools to develop a preoperative pathway incorporating prehabilitation for cancer patients preparing for surgery	N = 27 gynaecological and thoracic cancer patients Mean age – 70 years	Cohort study	All participants were approached at least 3 weeks prior to surgery Baseline function of those referred to the prehabilitation programme were below age-related normal values 6-min walk test = 301 m 5- times sit-to-stand = 12.4 s Dynamic gait index score = 20.1	Strengths: use of validated screening tools to identify patients suitable for prehabilitation Limitations: Of 27 patients referred for prehabilitation, only 21 patients were actually seen for intervention due to scheduling conflicts
Prehabilitation in cancer care: patient’s ability to prepare for major abdominal surgery 2021 [38]	Denmark	To investigate what patients with abdominal cancer due for surgery were able to do when provided with multimodal prehabilitation recommendations on physical activity, nutrition, psychological wellbeing, smoking cessation, alcohol cessation and preparedness for surgery	N = 30 ovarian cancer patients Mean age- 60 years	Mixed methods: Quantitative- participants were asked to track their progress on a diary using tick boxes and free-text Qualitative- Semi-structured interviews	Greater than 50% patients adhered to over 75% of recommendations on the prehabilitation leaflet provided Exercise significantly increased by 34% in the ovarian cancer group. Preferred exercises were walking and practical activities that helped preparedness. These activities may not necessarily increase heart rate in the way the recommendations had suggested Number of days with activity ranged from 1–18 days Feeling too unwell to participate was a significant barrier for over 60% of patients None of the smokers successfully stopped smoking	Strengths: Mixed methodology provided understanding of adherence to prehabilitation recommendations and follow up with semi-structured interviews shed light on what was acceptable as well as the barriers to participation Limitations: All data was self-reported so there was a risk of over-reporting amongst participants Interview follow-up with was with a limited number of people n = 5, mixed cohort (ovarian and colorectal) The interviewer and participants had previously met and the participants were aware that the interviewer was involved in designing the leaflet No considerations made about how to improve the smoking cessation aspect of the programme
What matters to you? An investigation of patients’ perspectives on and acceptability of Prehabilitation in major cancer surgery 2021 [39]	Denmark	To understand perspectives on and acceptability of prehabilitation among patients undergoing abdominal cancer surgery by providing them with a leaflet with prehabilitation recommendations around physical activity, nutrition, psychological wellbeing, smoking cessation, alcohol cessation and preparedness for surgery	N = 12 ovarian ca patients	Mixed methods- quantitative and qualitative Cohort study + semi-structured interviews	The preoperative period: Participants expressed readiness and prehabilitation was deemed feasible. Still had to the capacity to ‘act’ despite several pressures they were facing Short time frame between diagnosis and treatment was a major concern. Prehabilitation is less of a priority In the stressful time, doing meaningful things such as meeting friends/family, work and everyday tasks seemed more important ‘Last chance to live normally’ Attitudes towards prehabilitation: Prehabilitation is beneficial but it needs to fit in to their everyday lives. Need a flexible and “tailor” made plan according to physical/environmental context Motivation for action: The need to ‘report’ activity to healthcare professionals was motivating. Also, the ability to choose their activities meant reduced likelihood of failure The need for support: Whilst freedom and flexibility were important, there was a strong need for guidance and close contact with healthcare professionals Suggestion that facility-based programmes would be more successful however most preferred at home-based interventions due to safety and convenience	Strengths: Patients were interviewed following a trial of written advice (not totally naïve) The generalised recommendations in the leaflet allowed participants to tailor their preparation according to themselves and their everyday lives Limitations: the general recommendations could be considered too vague or irrelevant Homogenous and Dutch speaking sample only, which does not represent a wider, more representative population
Investigating the experiences, thoughts and feelings underlying and influencing prehabilitation among cancer patients: a qualitative perspective on what, when, where, who and why 2020 [40]	Denmark	To investigate thoughts, experiences, feelings of prehabilitation prior to major abdominal surgery by providing participants with a leaflet of recommendations around physical activity, nutrition, psychological wellbeing, smoking cessation, alcohol cessation and preparedness for surgery	N = 7 ovarian cancer patients Median age- 58 years	Mixed methods: Quantitative- participants were asked to track their progress on a diary using tick boxes and free-text Qualitative- Semi-structured interviews	What: Prehabilitation is not the only way to prepare for surgery. Participants would rather prepare for life and death. Meal preparation, house cleaning, laundry, gardening, writing a will, funeral planning, reviewing insurance were examples of prioritised activities When: Pre-operative period considered both ‘too short’ and ‘too long’ Short time considered positive, meaning patients would be on the other side sooner. However, also considered too short to complete all the tasks they need to do. Of which, prehabilitation was not considered a priority. Some felt that prehabilitation should be introduced earlier. Some suggested delaying treatment but all patients eluded to wanting surgery done sooner rather than later Where: Patients appreciated home-based recommendations Physical symptoms e.g., fatigue, nausea, vomiting and diarrhoea easier to manage at home Psychological issues stopping people leaving the house Able to fit around everyday lives, work, home tasks and family life Already spend too much time in hospital with appointments Travelling to and from hospital is time-consuming d based interventions were potentially more motivating with likely greater chances of success and adherence Support from healthcare professionals and other patients would be an opportunity for ‘community’ and social interaction Who: Prehabilitation was considered unsuitable for those who are either too fit or unfit Relatives considered supportive but patients didn’t want to burden them, hence friends and colleagues more crucial support system More involvement by healthcare professionals requested to force, threaten and encourage/motivate patients to be involved. Could lead to some resistance though if felt pushed Why: Having to fill out a prehabilitation diary was motivating and patients felt obligated to do so Motivated by the positive health outcomes of engaging with prehabilitation i.e., strength body, feeling calm and early discharge	Strengths: All opinions surrounding ‘what’ and ‘when’ and ‘who and ‘why’ were based on real experience with the leaflet Limitations: Relatively young population- not translatable to elderly but highlights issues that even younger patients experience All opinions on ‘where’ were hypothetical
Advanced ovarian cancer patients identify opportunities for Prehabilitation: A qualitative study 2021 [41]	USA	Investigate potential barriers and facilitators of engaging with prehabilitation during neoadjuvant chemotherapy	N = 15 advanced ovarian cancer patients Mean age -64 years All received chemotherapy over 6–8 cycles	Qualitative – In depth interviews	Physical activity during neoadjuvant chemotherapy: 11/15 participants reported not taking part in structured exercise during chemotherapy at baseline. 14/15 reported continuing activities of daily living 93% of participants were willing to take part in structured exercise during chemotherapy even if they had not done so prior to diagnosis 3–7 days per week, 15-30 min per day of walking, strength training, yoga/stretching was considered acceptable Barriers to structured physical activity: Physical symptoms e.g., fatigue, difficulty breathing, abdominal pain/distension (cancer related), nausea and vomiting, neuropathy, and bone pain (treatment related) Access/social barriers: Distance from home, money, time, needing to work full time Psychosocial barriers: Disengagement with society- feeling low, baldness, not going to the shops to buy groceries Motivators to structured physical activity: The perception of improved overall health and wellbeing i.e., physical and mental. Ability to engage with grandchildren Improvement in cancer related outcomes i.e., surgical outcomes and prognosis Influence of community and providers: support system to encourage and motivate exercise, instructions by healthcare professionals	Strengths: Specific to barriers and facilitators to functional optimisation prior to surgery were highlighted through in depth, rich data from interviews Limitations: Non-diverse cohort (homogenous for race, ethnicity, socio-economic status and language) Prehabilitation naïve and not given information prior to being interviewed No information on education status/employment or living situation
PRE-surgery thoughts- thoughts on prehabilitation in oncologic gynaecologic surgery, a qualitative template analysis in older adults and their healthcare professionals 2021 [42]	The Netherlands	To investigate possible content and indications for prehabilitation and potential barriers amongst gynaecologic cancer patients and their healthcare professionals	N = 16 patients with a high risk of gynaecologic malignancy Mean age- 70 years N = 20 multidisciplinary professionals- clinical nurse specialist, oncologists, surgeons, allied health professionals	Qualitative -Semi-structured interviews	Thoughts on prehabilitation: Overall positive reaction towards prehabilitation. Patients assumed a positive benefit whilst professionals felt the need to ensure it was evidence based Facilitators: Motivational reasons: Urgency, sense of control, self-efficacy, doing something positive Motivational support: Patients appreciated support through activity trackers, pedometers, and diaries. Human support from family/friends, community and professionals considered crucial too Practical facilitators: Prehabilitation should be part of a routine and encouraged by a motivated and dedicated team Barriers: Patient: Stress (too many appointments), physical condition, lack of knowledge, limited access to digital resources, language barrier Patient practical factors: Travelling to hospital for prehabilitation, time between diagnosis/surgery (as little as 1 week) and negativity surrounding postponement Organisational practical factors: Financial implications, lack of capacity, too much on the gynaecologist, lack of evidence base, lack of knowledge, lack of coordination Suggested model: Screening to be carried out by a physician assistant or nurse specialist. If fit for surgery, then general advice. If not, then referred to specific advice or referral to the multidisciplinary team with nursing support throughout being pivotal to success	Strengths: Convenience sampling followed by purposive sampling for diversity in age, educational level, diagnosis, and physical condition for patients Variety of professionals from multidisciplinary team (except psychologists) from district general and teaching hospitals Interviewer had extensive experience in qualitative research Limitations Patients only provided with a brief of prehabiliation and did not undergo the intervention themselves. Therefore, all answers relating to prehabilitation directly are hypothetical
Enhanced recovery after gynaecological/oncological surgeries: Current status in India 2020 [43]	India	Establish peri-operative practices performed by several gynaecological and oncological surgeons in India	N = 100 responses: N = 83 surgical oncologists N = 17 gynaecological Oncologists across 59 different institutions in India	Online cross-sectional survey	100% of respondents educated patients with pre-admission information and counselling prior to surgery 60% educated patients through oral and written communication 37% oral communication only 98% advised prehabilitation Of which 71% advised trimodal approaches 15% advised nutrition only, 12% exercise only and 1% anxiety only 53% advised starting prehabilitation at the time of planning surgery and 42% earlier at the first outpatient department	Strengths: Relatively large number of respondents, multi-site and across specialties Limitations: Limited description of the prehabilitation programmes which are recommended or provided and the respective outcomes Survey was limited to gynaecological and surgical oncologists with no input from the multidisciplinary team
Enhanced recovery after surgery (ERAS) in cytoreductive surgery (CRS) and hyperthermic intraperitoneal chemotherapy (HIPEC): A cross-sectional survey 2021 [44]	India	To capture clinicians’ practices about ERAS (including prehabilitation) in patients undergoing CRS or HIPEC	N = 136 Surgical oncologists, anaesthesiologists, gynaecological oncologists and intensivists	Online cross-sectional survey	The respondents recommend/practice the following: Perform incentive spirometry and corrected anaemia- 94% Smoking cessation- 82% Review alcohol consumption- 80% Encouraged exercise- 76% Recommend immunonutrition- 24% Psychological component considered a ‘non-essential’ part of the working ERAS protocol within prehabilitation	Strengths: Insight in to practices amongst clinicians working across India and specialties Limitations: Did not include programmes or description of facilities available to support Lack of allied health professional involvement
Prehabilitation for medically frail patients undergoing surgery for epithelial ovarian cancer: a cost effectiveness analysis 2021 [45]	USA	To assess potential cost-effectiveness of prehabilitation in patients undergoing surgery for ovarian cancer Based on the hypothesis that nutrition, functional status, medical co-morbidities, mental health, and social situation all impact frailty Frailty is a key contributor to post-operative complications, increasing length of stay, increased non-home discharges and discharge to care facilities	N = 4415 women with ovarian cancer Estimated based on figures at 66–80% of 22,530 patients diagnosed undergo PDS. Of which 24% are frail according to Mayo clinic. Produces approx. 4,400 patients	Cost-effectiveness analysis	For a cohort of 4415 women: Usual care costs $404.9 million whilst prehabilitation is cost saving at $371.1 million Per patient, cost saving = $9,418 Tornado analysis found that the greatest contributors to the Incremental Cost Effectiveness Ratio of 100,000 dollars per life per year were as follows: -90- day mortality after complication with usual care (0.97) -90-day mortality after complication in those receiving prehab (0.31) -Surgical complication after prehab (0.33) -Surgical complication after usual care (0.21)	Strengths: Only cost-effectiveness analysis in the ovarian cancer cohort Limitations: Theoretical model based on model inputs (limited by their individual precision)- requirements for larger and more prospective trials Cost effectiveness based on care and nursing home residence in Ohio
Role and Impact of multimodal prehabilitation for gynaecologic oncology patients in an Enhanced Recovery After Surgery (ERAS) programme 2019 [46]	Spain	To review the literature surrounding prehabilitation for gynaecological cancer patients and accordingly suggest a safe and reproducible multimodal prehabilitation model for gynaecologic cancer patients that can be tested in various centres	N/a	Review and proposal of a multimodal prehab model based on current literature	3 evaluation time-points: -Baseline: 2–4 weeks prior to surgery (screening and referrals) -Pre-operative: 1 week prior to surgery -Post- operative: 8 weeks All participants to record on diary which will evidence compliance All participants fill out SF-12^•^ at each time point Baseline assessment to be carried out thoroughly by consultant and anaesthetist Medical optimisation: Identify and manage comorbidities, stop tobacco and alcohol consumption, hospital pulmonary programme, anaemia- iron correction, frailty- referral to geriatrician, poor social situation- referral to social assistant Physical Activity: 6MWT and VO_2_ max is calculated. If VO_2_ max < 12, patient undergoes supervised physiotherapy programme If VO_2_ max > 12 given home based exercises with aerobic, flexibility and respiratory training Everyone advised inspiratory exercises 10 min every 8 h and mobilisation in hospital as soon as possible Nutritional Intervention: MUST^¥^ screening and albumin If MUST < 2 general advice to increase calories. MUST > 2 and albumin < 3, patient gets an individual dietary plan and oral nutritional supplements. Everyone is given a recipe book for protein shakes and meal planning Feeding is commenced as soon as possible post-operatively Psychological Intervention: Assess through HADS^≠^ (total score 21). Score < 7 advised general relaxation and breathing exercises 20 min prior to lunch and dinner. Score > 7 Referral to psychologist. Everyone encouraged to attend free mindfulness session once/week	Strengths: Produced a rigid and descriptive model with time points, treatment pathways and outcome measures Limitations: Fully hypothesised programme based on theoretical evidence Yet to have published outcomes from a trial of this model

ERAS* = Enhanced recovery after surgery, 6MWT** = Six-minute walk test, BMI^↓^ = Body Mass Index, NACT*** = Neoadjuvant chemotherapy, VO_2_ max^$^ = Maximum oxygen consumption, SF-36^ = 36-Item Short Form Survey, SQUASH^£ =^  = Short QUestionnaire to ASsess Health enhancing physical activity, PG SGA-SF^#^ = Patient Generated Subjective Global Assessment Short Form, SF-12̏· = 12-Item Short Form Survey, MUST^¥^ = Malnutrition Universal Screening Tool, HADS^≠^  = Hospital Anxiety and Depression Scale

**Table 3:** Description of the multimodal prehabilitation interventions using a modified version of the TIDieR checklist

Study	What		Who Provided	How	Where	When and how much	Tailoring	How well (actual/planned)
	Components	Description						
SOPHIE trial (Randomised controlled trial PROTOCOL) [23]	-Exercise -Nutrition -Psychology No theory base reported	ERAS* in addition to- Physical activity: high intensity endurance exercise and physical activity promotion. Nutrition: Counselling to achieve 1.5–1.8 g/kg of protein in addition to whey supplementation. Psychological: motivational interviewing, mindfulness and cognitive behavioural therapy	Information not provided	Physical activity data and promotion is remotely controlled using computer technology. No further information provided on this or how the nutrition and psychological components are delivered	Information not provided	Information not provided	Information not provided	The following outcomes are measured up to 30 days post-operatively: Complications (Clavien-Dindo classification), Hospital and intensive care length of stay, compliance with ERAS using a checklist of items, cost effectiveness, aerobic capacity, health related quality of life, nutritional status (GLIM** criteria), cognitive deficit (WAIS***)
PHOCUS (Randomised controlled trial PROTOCOL) [24]	-Exercise -Nutrition -Psychology No theory base reported	Physical activity: Functional capacity measurement and consultation. Nutrition: Consultation, malnutrition scoring and dietary supplements. Psychological: Consultation, psychological support and anxiety and depression scoring	A rehabilitation specialist, nutritional specialist and clinical psychologist are responsible for delivering the respective components	Information not provided	Information not provided	Information not provided	Information not provided	Functional capacity changes- 6MWT^¥^ at 9–12 weeks post-operatively
Home-based telemonitoring prehab for major abdominal surgery (Randomised controlled trial PROTOCOL) [25]	-Exercise -Nutrition -Psychology No theory base reported	Physical activity: All participants undergo a baseline functional assessment (details not provided) They are then provided with an actigraph to monitor their daily step count and sedentary time. Nutrition: All participants undergo a baseline nutritional assessment (no details provided). Psychological: All participants undergo a baseline QOL assessment	Daily step information and sedentary time recorded by the actigraph is sent to a Registered Nurse in real time via the app, when pre-determined thresholds are met. The nurse will contact the participants over the telephone. Surgeons are also able to communicate with caregivers and patients	The nurse communicates with the patient via the TapCloud app and rings them if required. Further face to face or virtual focus groups can take place between the surgeon, caregivers and patients	Participants will undertake daily step counts in their own time and chosen location. No information is provided about where the baseline assessments or face to face focus groups will take place	The actigraph measures daily physical activity. The programme begins prior to surgery and the step counting continues up to 14 days post-operatively	Prior to beginning the programme, participants undergo a home assessment (no details provided) and according to the findings, a tailored prehabilitation programme is set up for them	Change in daily step count (Baseline up to day 14). Post-operative complications using Clavien-Dindo classification (Up to 30 days after surgery). Time to hospital readmission (up to 3 months). The following are measured up to 4 months after surgery: Qualitative data from exit interviews, time to early withdrawal, change in sleep, change in general symptoms, change in sedentary time
Prehabilitation plus ERAS vs ERAS in gynaecological surgery (Randomised Controlled Trial PROTOCOL) [26]	-Exercise -Nutrition -Psychology No theory base reported	All participants will undergo physical activity, nutrition and psychological counselling in addition to ERAS. Not details on the individual components provided	The individual components are overseen by the multidisciplinary team but no specific details provided	Information not provided	Information not provided	Information not provided	Information not provided	The following are measured up to 30 days post-operatively: Patient readiness for discharge (ability to walk independently, take care of herself and eat 75% of her required calories). Surgery related complications (Clavien-Dindo classification), Hospital readmissions, ICU admissions, Health related quality of life. The following are measured up to 60 days: Change in body mass (bioimpedence analysis), change in muscle strength (dynamometer), functional capacity (6MWT) and HADS^•^
Connected Prehabilitation program during NACT (Randomised Controlled Trial PROTOCOL) [27]	-Exercise -Nutrition -Psychology No theory base reported	Physical activity: Standardised preoperative physical activity. No details on intervention provided. Nutrition: Care in line with local guidelines. Psychological: Support with coping strategies	Dietitian responsible for nutrition intervention. No information provided about who is responsible for delivering physical activity and psychological interventions	One to one supervision via an app which connects to a smart watch and scales	Multi-centric trial involving 7 cancer care centres or university hospitals. The individual components of the programme are home-based	Information on how frequently patients will undertake individual components is unknown	Exercise and nutrition goals tailored to the participant depending on the recordings of the fitness watch and scales	The following will be measured at baseline, prior to surgery and 3 months post-operatively: V0_2_ max, IPAQ^↓^, muscular strength using dynamometer, HADS, cancer related quality of life using QLQ-C30 $\infty$, BMI, weight, muscle mass by computed tomography, surgical morbidity rate using Clavien- Dindo classification
Gyn Onc Prehab study (Randomised controlled trial PROTOCOL) [28]	-Exercise -Nutrition -Psychology Not based on a formal model but based on theory that older patients are at higher risk of deconditioning post-operatively and the arduous journey of cancer treatment will adversely impact mental health	Physical activity: Completion of 6MWT, grip strength and time up to go test. Nutrition: Completion of Patient Generated Subjective Global Assessment and targeted questioning by the dietitian. Psychological: Quality of Life FACT-G^€^ questionnaire	Dietitian responsible for nutrition intervention. No information provided about who is responsible for delivering physical activity and psychological interventions	One to one physical activity and nutritional interventions. Group psychological counselling	No information provided about where the interventions will take place	Each component will be delivered to the participants pre-operatively (by approximately 4 weeks) and at 4 and 8 weeks post-operatively. Total 12 week study period	Due to the urgency of some diagnoses, surgeries will not be delayed and therefore, some participants may not complete all components of the study	The following will be measured at the end of the 12 week study period: 6MWT, grip strength and time up to go, readmission rate, complication rate, patient satisfaction (anonymised questionnaire), Quality of Life FACT-G assessment and treatment completion
PROADAPT- ovary/EWOC-2 (Randomised controlled trial PROTOCOL) [29]	-Exercise -Nutrition -Other Based on logic change model, constructed with literature data and validated by an expert group through a DELPHI method: the rehabilitation model	Pre-operative: Physical activity- strength training, endurance and breathing exercises. Nutrition- education and activity but no further details provided. During hospital recovery: Implementation of a standardised protocol within the MDT and pharmaceutical reconciliation. Post-operative hospital to home discharge activity	No information provided	Follow up will take place over the telephone. No information provided on whether the interventions are remote, face-to-face, and one-to-one or group based	The post-operative recovery and discharge initiatives are presumed to be hospital based	Phone call once a week, for 12 weeks, followed by once a month	Information not provided	30 and 90 day morbidity post-operatively using Clavien-Dindo classification. Completion of cytoreductive surgery and 6 cycles of chemotherapy within 2 years. Progression free and overall survival in 2 years
Training-Ovary 01 (Randomised Controlled Trial PROTOCOL) [30]	-Exercise -Nutrition -Psychology Based on the hypothesis that prehabilitation during neoadjuvant chemotherapy will produce a fitter patient prior to surgery and reduce treatment morbidity, mortality and improve oncological outcomes	Participants receive connected devices (watch, body fat weight scale) and have an application installed on their smartphone, allowing them access to the individual components of the programme Physical activity: training programme through short videos Nutrition: Advice provided in line with ESPEN guidelines Psychology: Coping strategies	A dietitian will provide nutrition support No information provided on who will oversee the exercise and psychology components	All participants will have their exercise and body composition data transmitted to the care team via the smartphone application Supervision takes place by the connected devices	The programme is entirely remote	Participants are recommended to perform exercise daily Nutrition support is adapted based on weekly body composition measurements No information provided on the frequency of using coping strategies The programme will be delivered over 3–6 cycles of neoadjuvant chemotherapy	Nutritional advice and exercise are tailored to the participants’ activity, chemotherapy regimen, weight variation, fat and lean body mass	Change in the VO2max between baseline and surgery for those who received prehabilitation compared to those who did not Up to 3 months post-operatively: Global Physical Activity Questionnaire, muscular strength (brachial biceps), psychological status (HADS, cancer-related quality of life (QLQ-C30), motivation (unstructured interviews) Nutritional status: weight, BMI, muscle mass Morbidity (Clavien-Dindo score), hospital length of stay, mortality rate, readmission Mortality: overall survival, disease free survival Cost effectiveness
F4S PREHAB Trial (Stepped-wedge cluster randomised controlled trial PROTOCOL) [31]	-Exercise -Nutrition -Psychology -Other No theory base reported	All participants undergo the following screening within each component: Physical activity (SQUASH ^±^ questionnaire, submaximal Astrand test, indirect 1RM, steep ramp test) Nutritional intervention (length, body weight, fat-free mass, PG-SGA SF^×^), Psychological support (SF-36‣). Smoking cessation support. No details of individual interventions provided	No information provided	Information not provided	Information not provided	Screening will take place 4 or 8 weeks prior to surgery. Follow up tests will take place 1 week prior to surgery. No information is provided on when and how often participants undertake the individual components of the intervention	Information not provided	1 month post-operatively: Length of stay, post-operative complications (Clavien-Dindo score and Comprehensive Complication Index Score).3–6 months post-operatively: Quality of life questionnaires (SF-36 questionnaire + iMCQ questionnaire + EQ-5D-5L questionnaire), Physical activity (SQUASH questionnaire)
Impact of remote prehabilitation programme (Retrospective cohort study ABSTRACT) [32]	-Exercise -Nutrition -Psychology -Other No theory base reported	Physical activity: physiotherapy input Nutrition: dietetics input Psychology: Psychological help Other: Early anaesthetic input. No details provided on the individualised components	Physiotherapists, dietitians, psychologists and anaesthesiologists were responsible for delivering the respective components	The programme was 'remote' No details provided on how the individual components were delivered	All components were delivered remotely and therefore, facilities were not required	Information not provided	Based on the above study, all components were delivered remotely and participants were not expected to attend the hospital	Over a 12 month period, 25 participants enrolled for prehabilitation. 100% had early anaesthetic and physiotherapy input. 48% needed dietetics 44% took up psychological help. In prehabilitation group, only one patient had their surgery delayed, compared with 6 patients did not receive prehabilitation
The Royal Mile (Prospective cohort study ABSTRACT) [33]	-Exercise -Nutrition -Other No theory base reported	Physical activity: Home-based exercise based on Macmillan Move More home exercise pack and advice. 20% of participants offered circuit training as a limited resource Nutrition: Participants underwent urgent dietetic review if malnourished and were given oral supplementation if they scored > 10 on the Royal Marsden nutrition Screening Tool. All participants were given a Macmillan Healthy Eating and Cancer pack and nutritional advice Anaemia management: All participants had baseline iron, folate and vitamin B12 measurements and if low, were treated as per local protocol	The clinical nurse specialist provided physical activity and nutrition advice. A dietitian carried out urgent reviews for anyone who was considered malnourished according to local screening tool. No information provided on who was meant to deliver circuit training	No information provided on whether the consultations with the nurse specialist and dietitian were face to face or virtual	Circuit training was designed to be hospital based. No information provided on whether this was an individual or group based session. No information provided about where the dietetic and nurse consultations took place	Information not provided	All patients scoring > 10 on local nutrition screening tool were seen by a dietitian and prescribed oral supplementation. Those who were anaemic received treatment only	50% of patients were anaemic and 28% received iron. 30% patients were malnourished. 3/18 patients were asked to attend hospital based exercise but zero attendance due to travel, pre-existing and cancer related comorbidities
Prehab Matters (Prospective cohort study- ABSTRACT) [34]	-Exercise -Nutrition No theory base reported	Physical activity: baseline assessments: 6MWT, SF-36 questionnaire, complete physiotherapy assessment followed by individualised exercise plan and invitation to supervised exercise classes Nutrition: Full nutritional analysis followed by individualised dietetic plan	No information provided on who was responsible for screening the participants. Each participant underwent assessments by Physiotherapists and Dietitians. No information on who led the exercise classes	Baseline assessments took place face-to-face or over the telephone. Exercise classes were offered to be group based or individualised	Exercise classes were hospital based	No information is provided on the length of the prehabilitation period. Participants were invited back 6 weeks post-operatively to have baseline measurements repeated	If distance to the hospital was a barrier for attending face to face consultations, participants were offered a telephone consultations. One-to-one or group sessions offered for exercise classes	Of 142 participants who underwent a baseline assessment, 26 were telephone and 116 were face to face. 28 participants took part in exercise classes and attended a median of 3 classes attended per person (range 1–14) for the 116 people who chose classes. Of 142, only 33 attended post-surgery follow up. 12/32 patients suffered a complication post-surgery. Median length of stay was 6 days. At 6 weeks follow up, patients maintained BMI, restored SF-36 quality of life and significantly improved 6MWT. Participants reported that the programme made them feel 91% more able to cope with surgery. A survey found that participants and their families are both more likely to make long term lifestyle changes as a result of attending the programme
Prehab to enhance post-operative recovery for an octogenarian following hysterectomy (Case Study) [35]	-Exercise -Nutrition -Psychology No theory base reported	Physical activity: Baseline assessment: 6MWT. Intervention: Strengthening of upper extremities (shoulder flexion, horizontal abduction, shoulder blades squeezing, seated row, biceps and triceps curl). Lower extremities (hamstring curls, ankle pronation, static quads, bridging, hip abduction exercises) Breathing/Cardiovascular (abdominal breathing, ambulation 15–20 min after 60 min rest Nutrition: Baseline assessment: Serum albumin and 24 h dietary recall. Intervention: Encouraged to increase kcal and protein intake and a daily supplement of 30 g soy kefir Psychology: Baseline assessment: SF-36 and RBANS^‡^	Kinesiologist, dietitian and, psychologist were responsible for delivering the respective components	The intervention took place face-to-face, on a one-to-one basis with the participant	The intervention took place in the participant's home	The intervention took place in the 3 weeks prior to surgery. The exercise component took place 3 times per week, 1 h each session. The kefir supplementation was encouraged daily	The interventions were tailored based on the outcomes of the baseline assessments	Post-operative measures at 6 and 8 weeks: Improvement in 6MWT but below normal average, marginal dietary improvement but suboptimal energy and protein intake. Improvement in physical and mental components of SF-36. Psychologist observed improvements and self-reported improvement in mood and concentration attributed to physical activity and visits by the kinesiologists
Sarcopenia in surgical oncology patients referred for prehabilitation (Retrospective cohort study) [36]	-Exercise -Nutrition -Psychology No theory base reported	Physical activity: Baseline assessments: Evaluation of musculoskeletal or neuromuscular conditions with relevant treatment. 6MWT, grip strength, 5STS^⊲^ Intervention: Participants received individualised exercise programmes modelling American College of Sports Medicine and American Cancer Society’s exercise recommendations for cancer survivors. Advised to engage in 30 min of moderate intensity exercise, 3–5 days per week, including 2 sessions of body strengthening exercises Nutrition: Baseline assessment: Body composition using a dual frequency total body bioimpedance scale. No information provided on intervention Psychology: Stress and anxiety management. Motivation	Registered nurse responsible for coordinating and educating participants about the programme and monitoring adherence to recommendations A senior physiotherapist undertook baseline functional assessments and deliver demonstrations of exercises A counsellor led the psychological component	Nurses followed up with participants over the telephone. If participants completed reported more than 150 min of aerobic activity and at least 2 strengthening activities, per week, they were considered fully adherent Exercise demonstrations were delivered by physiotherapist in person. Videos and written recommendations were provided to allow participants to carry out physical activity at home No details provided on how participants interacted with the counsellor	Baseline and outcome measures were taken in person, at the dedicated clinic The exercise intervention was home-based No information provided on where the psychological component took place	No information provided	Exercises delivered by the physiotherapist were subject to tailoring as per participant ability	Adherence to exercise programmes: 32% full, 25% partial, 17% no adherence and 25% unknown Taking part in prehabilitation significantly improved 6MWT and 5STS. The duration of prehabilitation had no significant effect on 6MWT
Implementing prehabilitation as part of ERAS (Prospective cohort pilot study ABSTRACT) [37]	-Exercise -Nutrition -Psychology No theory base reported	All participants were screened using the FRAIL index and Centers for Disease Control and Prevention fall risk screening tool. If score > 2, referred for prehabilitation Physical activity: Baseline assessments: 6MWT, 5STS and dynamic gait score. Intervention: Individualised exercise programme. No details provided Nutrition: Individualised nutrition programme. No details provided Psychology: Screening for mood impairments. No details of psychological intervention provided	No information provided on who was responsible for delivering the components of prehabilitation	Information not provided	Information not provided	All participants were screened at least 3 weeks prior to surgery	Information not provided	27 referrals were received for prehabilitation. Average age was 70 years. Baseline functional status was below age-related normal values No information provided on the impact of prehabilitation on treatment outcomes
Patient's ability to prepare for major abdominal surgery (Prospective Cohort Study) [38]	-Exercise -Nutrition -Psychology -Other Based on 'The Complex Interventions Framework' developed by the Medical Research Council	All participants provided with a leaflet of recommendations- Physical activity: Participants encouraged to undertake exercise beyond day to day activities, which increase the heart rate. Examples provided Nutrition: Recommended protein rich diet, examples of high protein foods and protein shakes provided. Relaxation: Participants were encouraged to set aside time for relaxation as well as meditation and deep breathing. An audio file with exercises was provided to help Smoking cessation: website links to stop smoking. Alcohol cessation: All participants advised to stop drinking prior to surgery if they consumed more than 6 units per day General preparation: Recommended practical activities to prepare for discharge e.g. gardening, cleaning the house, keeping a diary	Nurses at outpatient clinic provided patients with leaflet of recommendations at first surgical appointment	All participants were asked to complete a diary of their compliance with the prehabilitation interventions as they undertook them in their own time. Participants were asked to return completed diaries when they returned to hospital for their surgery	All recommendations provided on the leaflet allowed participants to undertake the prehabilitation programme in their chosen locations and time	The information leaflet was provided to participants between 7–14 days prior to surgery. Participants were encouraged to carry out exercise, relaxation and eat a high protein diet on a daily basis. At least 20 min of relaxation and meditation was advised. Participants asked to complete a diary, ticking when they had completed recommendations. Also reporting symptoms and any barriers to achieving the recommendations. Completed leaflets were returned to the ward when patients arrived for their surgeries. Interviews then took place with participants to understand their thoughts, opinions, facilitators and barriers to engaging with prehabilitation	Participants were encouraged to complete everyday activities beyond exercise and relaxation which would help them prepare for the post-operative recovery period. The remote nature of the programme and generic recommendations allowed patients to choose activities and foods which were suitable for them	46 ovarian cancer patients received the leaflet, 37 agreed to participate and 33 returned completed leaflets. On average, they made notes/ticked boxes for 9 days prior to surgery. Number of days with activity ranged from 1–18 days. More than 50% adhered to more than 75% of recommendations Increased weekly exercise by 34% with walking as the most popular activity. 76% reported preparing in 'other ways': housekeeping, gardening, bag packing, preparing food, taking vitamins and socialising. 64% experienced feeling too unwell as a barrier to participation at some point. None of the participants drank more than 6 units of alcohol per day

ERAS* = Enhanced recovery after surgery, GLIM** = global leadership initiative on malnutrition, ***WAIS = Wechsler adult intelligence scale, 6MWT^¥^ = 6- minute walk test, HADS· = hospital anxiety and depression scale, V0_2_ max = maximum oxygen capacity, IPAQ^↓^ = international physical activity questionnaire, QLQ-C30 $\infty$ = quality of life questionnaire for cancer patients, FACT-G^€^ = functional assessment of cancer therapy- general, SQUASH ^±^ = short questionnaire to assess health enhancing physical activity, PG-SGA SF^×^ = patient generated subjective global assessment short form, SF-36‣ = 36-item short form survey, RBANS^‡^ = repeatable battery for the assessment of neuropsychological status, 5STS^⊲^ = five times sit to stand

## Results

**Fig. 1:**
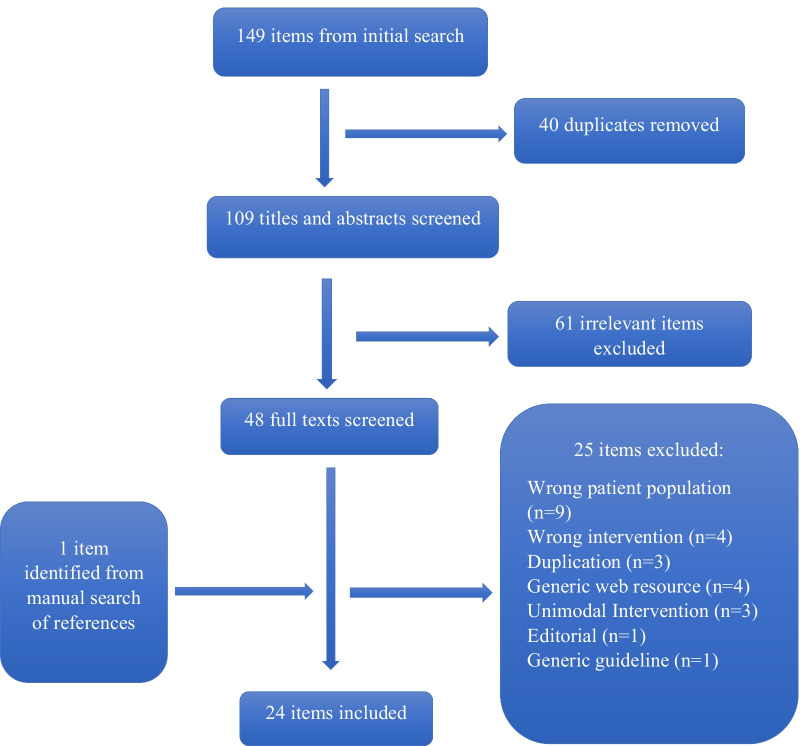
PRISMA diagram illustrating the process by which articles were selected for inclusion

**Fig. 2:**
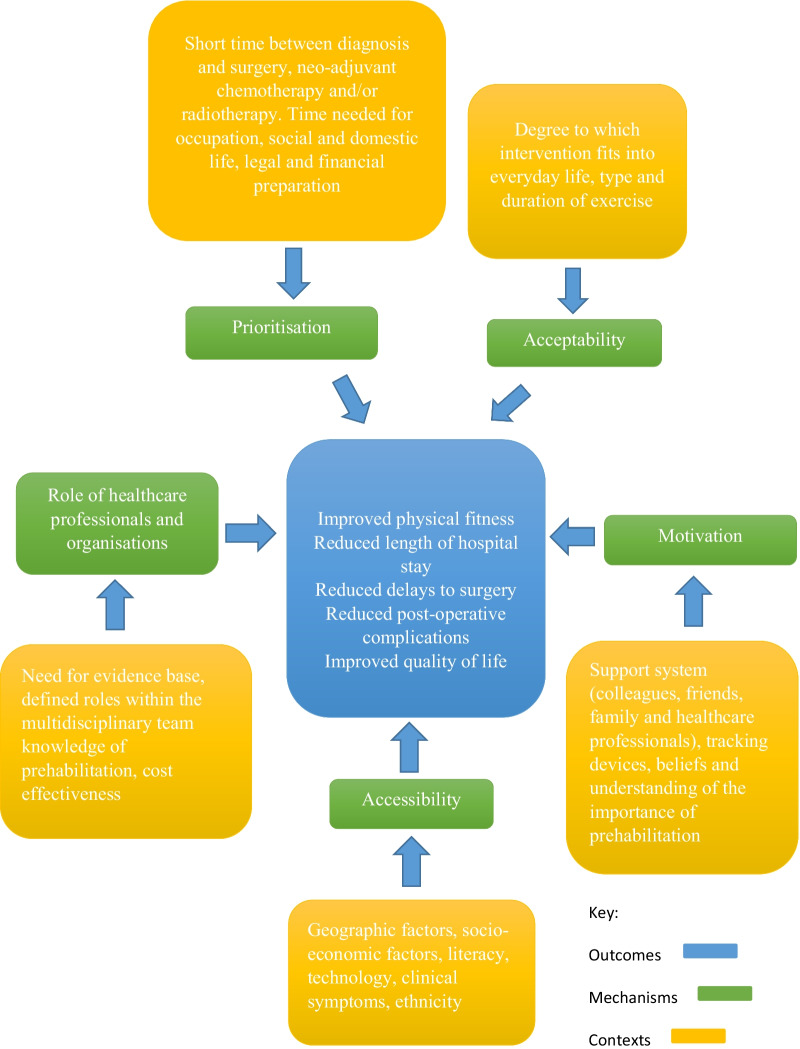
The contexts and mechanisms influencing engagement with prehabilitation to achieve intended outcomes

In total, 24 studies were included in this scoping review (Fig. 1) and the results of the review are presented in narrative form, Tables 2, 3 and Fig. 2.

### Overview of studies

The 24 studies included nine registered protocols for randomised controlled trials of prehabilitation interventions [24–32], six observational studies (of which four were abstracts only) [33–38], three mixed-methods studies [39–41], two qualitative studies [42, 43], two cross-sectional surveys [44, 45], one cost-effectiveness analysis [46] and one systematic review [47] (Table 2).

The largest number of studies originated from the USA (n = 7). Other countries of origin were UK (n = 3), Denmark (n = 3), France (n = 3), India (n = 2), Spain (n = 2), Brazil (n = 1), Prague (n = 1), China (n = 1), The Netherlands (n = 1). UK data were limited to three abstracts describing pilot interventions [33–35], two of which related to ovarian cancer patients [33, 34], whilst the remaining abstract included only one gynaecological cancer patient in the study population [35]. The majority (n = 12) of studies included patients with ovarian cancer only. Six studies described their populations as ‘gynaecological cancers’ and five included a mixed group of ovarian, endometrial and vulvar cancers. One study included patients with endometrial cancer only. No relevant studies were found which included cervical or vaginal cancers. Sample sizes ranged from 1 to 194 gynaecological patients and the mean age range of participants was 58–88 years old, although some trials are open to patients from 16 years and above.

### How gynaecological cancer studies define prehabilitation

Prehabilitation interventions included in this review varied in their nature and duration. Our eligibility criteria excluded unimodal intervention studies e.g., those focussed on physical activity or nutritional optimisation only, but in fact these were also labelled as prehabilitation. Other studies described enhanced recovery after surgery (ERAS) programmes as prehabilitation. The authors of this scoping review agreed that ERAS is a separate intervention, although it might complement prehabilitation to provide effective pre-operative work up. Therefore, studies which included multimodal prehabilitation as a component of ERAS or in addition to it, were included in the present review, but studies referring to ERAS alone were excluded.

Sixteen studies described multimodal prehabilitation interventions. The three mixed-methods studies were from the same research group and described the same prehabilitation intervention, so these were considered as one study (Table 3).

These interventions all varied in terms of programme setting, nature and delivery of prehabilitation, participant criteria, duration of prehabilitation and measured outcomes. The reported duration of prehabilitation ranged from 2 weeks to 12 months, but most studies were unclear about the duration of intervention or contact time with health professionals in the prehabilitation period. Few studies provided a comprehensive description of all aspects of their prehabilitation intervention. Only two studies reported using theory to underpin the design of their complex intervention.

In terms of programme setting, most interventions adopted entirely remote supervision (n = 8) of which some were reliant on wearable technology and smartphone applications (n = 3). Some interventions were supervised face to face (n = 1) whilst others provided flexibility between facility-based supervision and remote supervision (n = 2). The remaining studies were unclear about the programme setting (n = 6). Only a few studies stated explicit involvement of a multidisciplinary team to deliver the individual components of prehabilitation (n = 8).

All programmes featured a physical activity and nutrition component (n = 16) and the majority of these also included a psychological component (n = 13). Interventions also included pharmaceutical reconciliation (n = 1), smoking cessation (n = 1), alcohol and smoking cessation (n = 1), anaemia management (n = 1) and pre-operative anaesthetic review (n = 1).

### Physical activity

Most of the interventions used screening tools to obtain baseline parameters for physical fitness, from which physical activity was prescribed. The 6- minute walk test (6MWT), a measure of mobility related function in older adults was commonly used. Other screening measures included grip strength, the maximum rate of oxygen the body is able to use during exercise (VO_2_ max), Five times Sit to Stand, The 5-item FRAIL scale (Fatigue, Resistance, Ambulation, Illnesses, & Loss of Weight), Short Questionnaire to Assess Health-Enhancing Physical Activity (SQUASH) and The International Physical Activity Questionnaires (IPAQ). Less than 50% of the interventions described their physical activity component in detail, beyond ‘physical therapy’ intervention or ‘exercise’. Of those which did, cardiovascular exercise to increase the heartrate, resistance training, circuit training and increasing daily step count were mentioned. Approved resources such as the Macmillan Cancer Support ‘Move More’ booklet were provided to all participants in one study.

### Nutrition

Screening tools were utilised by studies to assess for the risk of malnutrition, however, this was not as common as screening for physical function. Taking baseline anthropometry was the most common method of nutritional screening. Some studies utilised validated tools such as the Patient Generated-Subjective Global Assessment (PG-SGA), whilst a few used the Malnutrition Universal Screening Tool (MUST) or an adapted version of it. Interventions were commonly described as ‘dietetic consultation’ or ‘nutritional input, education or activity’. Two interventions made specific recommendations around increasing dietary protein and one intervention focussed on using a soy-based probiotic.

### Psychological wellbeing

Several validated tools were used to establish baseline psychological health and wellbeing amongst participants including the Short Form -36 questionnaire (SF-36) and Hospital Anxiety and Depression Score (HADS). However, the descriptions of psychological interventions ranged from being vague i.e., ‘psychological help’, ‘counselling’, ‘support’ and ‘coping strategies’ to more specific techniques like relaxation, mindfulness, cognitive behavioural therapy and motivational interviewing.

### The intended and unintended outcomes of participation in prehabilitation for gynaecological cancer patients

Interventional studies and protocols reported a wide variety of intended outcomes. These include improvements in physical conditioning and function (n = 10), post-operative complications (n = 7), quality of life (n = 7), nutritional status (n = 6), adherence to advice (n = 3), length of stay (n = 3), qualitative outcomes (n = 3), readmissions (n = 2), delays in surgery (n = 1), patient volume (n = 1), innate immune response (n = 1), cost-effectiveness (n = 1), sleep (n = 1), general symptoms (n = 1), progression free survival (n = 1) and overall survival (n = 1).

Only a few published studies reported actual outcomes of their prehabilitation programme, the majority of which were positive. Even fewer papers discussed unintended outcomes of their interventions i.e. adverse or surprising outcomes. In a UK based tertiary centre, multimodal prehabilitation delivered remotely during neoadjuvant chemotherapy to twenty five ovarian cancer patients led to a significant reduction (24–4%) in delays to major debulking surgery [33]. A case study of an octogenarian undergoing a hysterectomy for endometrial cancer found that a three week, tailored, multidisciplinary led, home-based prehabilitation programme improved her functional and mental capacity post-operatively but did not improve her overall nutritional status [36].

A mixed-methods study, in which thirty ovarian cancer patients were given written multimodal recommendations including exercises to increase the heart rate found that activity increased overall, mainly through walking and cycling [39]. However, many participants reported preparedness in additional ways to those recommended by the leaflet. For example, practical activities such as gardening, household cleaning, bag packing etc. Whilst this was not an intended outcome, the authors commented on the importance of encouraging tasks which contribute to the recovery period as a future consideration for prehabilitation programmes. In the same study, all participants were provided with information and resources on smoking cessation, however, none of the six smokers stopped smoking during the pre-operative period. Miralpeix et al. suggest the use of a hospital pulmonary programme, consisting of behavioural support and nicotine replacement therapy to support smoking cessation [47], however, this recommendation formed part of a theoretical model generated by the authors, the outcomes of which are not yet known.

### The key components, skills and contexts required by the healthcare team to implement a successful prehabilitation programme

Only four studies provided information about the components (e.g. guidelines, defined roles), skills (knowledge) and contexts (capacity and cost-effectiveness) required for healthcare teams. The only qualitative study to investigate the views of healthcare professionals found that clinicians value having a strong evidence base in order to advocate prehabilitation [43]. Defined roles for all members of the multidisciplinary team were also considered essential to streamline the process of prehabilitation. Oncologists in this study did not feel they had the capacity to oversee prehabilitation, therefore the authors presented a model in which the clinical nurse specialist was at the core of screening and triage, provided there were clear guidelines and screening tools available to support their role.

A cross-sectional survey of peri-operative practices amongst 100 surgical and gynaecological oncologists in India found that 98% of respondents advised prehabilitation, of which 71% recommended trimodal interventions (physical activity, nutrition and psychological input) [44]. In another survey of 136 Indian anaesthesiologists, gynaecological oncologists, and intensivists, 76% recommended preoperative exercise and even greater proportion recommended correction of anaemia, smoking cessation, and alcohol consumption. Immunonutrition was the least recommended intervention [45]. Interestingly, based on the survey responses, the psychological component of prehabilitation was considered non-essential. Only one study provided data from an organisational perspective. A cost-effectiveness study based on model inputs in the USA suggested that prehabilitation could potentially save over $9,000 per patient in a cohort of over 4,000 women [46].

### The facilitators and barriers to participating in prehabilitation amongst patients with gynaecological cancers

The existing evidence provides useful insights into key mechanisms and contexts acting as facilitators or barriers to engagement with prehabilitation. These can be summarised in the following themes: (1) Factors affecting patients’ views of the acceptability of prehabilitation (2) Factors affecting the motivation of patients to engage in prehabilitation (3) Prehabilitation as a priority (4) Access to prehabilitation.

#### Factors affecting patients’ views of the acceptability of prehabilitation

Very few studies directly explored the acceptability of prehabilitation from the patient’s perspective. Of nine protocols, only two documented their intention to assess acceptability and or satisfaction with the programme through exit interviews/questionnaires [25, 28]. However, all qualitative studies [26, 28], 32–34 reported something about the acceptability of prehabilitation from the patients’ perspective, mostly suggesting that patients are positive about engaging with prehabilitation due to the perceived and actual health benefits.

For a cohort of women in Denmark, prehabilitation was considered acceptable if it fitted in with their everyday lives and allowed them to carry out other tasks which helped them ‘prepare’ for surgery, such as meal preparation, laundry, gardening [40, 41]. Following the recommendations provided, women were ready to accept prehabilitation as being beneficial for health and wellbeing, but spending time with loved ones, funeral planning and finances were considered equally as important by some.

Ovarian cancer patients undergoing neo-adjuvant chemotherapy in the USA, were willing to engage in exercise despite lack of participation in structured physical activity at the point of diagnosis [42]. In depth interviews with those who were prehabilitation-naïve revealed that patients were theoretically willing to undertake 15–30 min of exercise on 3–7 days of the week. Activities such as walking, strength training and yoga/stretching were considered most acceptable.

Only one study commented on the acceptability of nutritional recommendations, in which patients felt that nutritional optimisation extended beyond the recommendations of a ‘high protein’ diet and should be more inclusive to fruits and vegetables [40]. Studies reporting baseline characteristics of participants found high rates of sarcopenia and malnutrition [33, 37, 38] and it is therefore unsurprising that nutritional components of prehabilitation are focussed around increasing protein intake [39, 47]. No studies specifically reported on the acceptability of psychological components.

#### Factors affecting the motivation of patients to engage in prehabilitation

Motivation appears to be a key mechanism influencing the engagement of patients with prehabilitation. Qualitative studies have revealed that patients believe that prehabilitation is beneficial to their health and wellbeing, treatment-related outcomes and cancer-related outcomes [39, 41, 42], and as such, these beliefs are motivating. Patients who participated in a UK based multimodal prehabilitation programme reported being more motivated to make long term lifestyle changes, as did their families [35].

The need for a support system to motivate patients was also identified. In three qualitative studies with ovarian cancer patients, support systems were available through colleagues, friends, and/or healthcare professionals [35, 42, 43]. However, one study found that some patients preferred not to ‘burden’ family members by relying on them, and therefore, identified healthcare professionals as the most appropriate motivators [41].

In addition to having a human support system, participants taking part in remote prehabilitation interventions identified progress tracking in the form of pedometers and diaries as highly motivating [40, 41, 43]. This supports the use of wearable devices in several of the trial protocols [31] whereby patients will have real-time fitness measures and outcomes that can be reported to healthcare professionals.

#### Prehabilitation as a priority

Another key factor influencing engagement is the degree to which patients prioritise prehabilitation, specifically in the context of time to treatment. Qualitative studies in Denmark and The Netherlands highlighted patients’ concerns around the short duration between diagnosis and surgery [41, 43]. With as little as a 1–2 week pre-operative period, patients felt the need to prioritise preparing for ‘life and death’ such a socialising, financial tasks and life administration.

A concern raised by patients was the large amount of time already spent in the hospital for appointments, and the possibility that prehabilitation programmes would require further attendance [43]. To address these concerns, van der Zanden et al. asked patients and healthcare professionals for their opinions on delaying surgery to allow for more pre-operative optimisation. They concluded that patients are unlikely to delay due to anxiety and a lack of evidence base supporting the decision to postpone surgical intervention. [43]. Several studies took advantage of three to six cycles of neo-adjuvant chemotherapy as a period for prehabilitation prior to surgery [31, 33, 34].

#### Access to prehabilitation

Qualitative findings suggested that prehabilitation needed to be locally accessible due to the cost of transport, appointment burden and limited pre-operative time [42, 43]. Several interventions included remote/home based interventions in their design [31, 33, 35, 39]. In the ‘Marsden Mile’ programme, initial results had revealed poor attendance to facility-based exercise [33], which led to the development of an entirely remote programme [34]. Lack of attendance to facility-based sessions was mainly due to ill-health, a finding highlighted in several other qualitative studies [41–43]. In these studies, patients suggested that their physical and mental health can act as barriers to engaging with society as well as the activities expected of them as part of prehabilitation.

No studies discussed whether ethnicity or age affected the accessibility of their prehabilitation interventions, although Polen De et al. did comment on the potential limitations of their entirely Caucasian cohort [41]. Three of the trial protocols excluded patients on the basis of poor understanding or inability to speak/write the primary language [32].

Figure 2 synthesises the contexts and mechanisms influencing engagement with prehabilitation. It illustrates the factors contributing to healthcare professional and patient engagement.

## Discussion

This scoping review aimed to summarise the quantitative and qualitative evidence for prehabilitation in women with gynaecological cancers, using a realist approach. To our knowledge, this is the first review to do this. We were already aware that there are no published trials for multimodal prehabilitation within gynaecological cancer [48], however our review provides a summary of several ongoing randomised controlled trials for which protocols have been published. Pilot observational studies suggest that prehabilitation is beneficial for this group, however, sample sizes of gynaecological cancer patients have been small and results are mostly limited to published abstracts [33–35, 38]. Our findings reveal several barriers and facilitators which need to be taken into account in future prehabilitation interventions for this group.

We acknowledge the limitations of this review. Firstly, it is possible that studies may have been missed by database searching as well those which were published after the search date. Secondly, this review only included studies with multimodal programmes involving more than one non-medical intervention, due to their perceived ability of meeting the complex needs of cancer patients. Therefore, studies reporting on unimodal prehabilitation programmes or those concentrating on medical management and optimisation, may have been missed.

Although descriptions of the interventions included in the scoping review are limited, our analysis of the contexts, mechanisms, and outcomes for prehabilitation provide useful insights into the factors that need to be considered in the design and implementation of prehabilitation for women with gynaecological cancer. It is now widely understood that the success of a complex intervention depends on the theory underpinning its design [44], which helps to explain the mechanisms underlying patient behaviour, based on what works for them and their circumstances [45]. Unfortunately, however, only two interventions found in the present study described the use of a logic change model [30] and framework [49] in their development. Moreover, few evaluated the acceptability of their interventions, despite this being an important consideration for complex interventions [50].

One study presented a working prehabilitation template for women undergoing surgery [47], and whilst it is detailed, flexible and plausible, it does not fully reflect the factors that might influence engagement with prehabilitation that we have identified. The qualitative literature in this field illustrates the complexity of delivering prehabilitation and sheds light on some of these factors. Our review suggests that both patients’ and healthcare professionals’ needs, views and respective roles must be considered in a successful prehabilitation programme. In order for healthcare professionals to engage with and deliver prehabilitation, they need a strong evidence base for prehabilitation within gynaecological cancer; defined roles for delivering prehabilitation within the multidisciplinary team and clear guidance around screening and triage of patients. Given that the existing literature does not yet provide strong evidence and clear guidance, engaging healthcare professionals may be challenging at this time.

The included studies suggest that patients value accessible prehabilitation services that are supported by a knowledgeable and motivated multidisciplinary team. Although it seems that surgical and gynaecological oncologists in some countries actively recommend prehabilitation as part of peri-operative management [29, 30], and that many believe it is important, there is a lack of awareness amongst professionals of the availability of prehabilitation services [49]. This suggests that there is still work to be done to educate the workforce around prehabilitation and to develop effective referral pathways between primary and secondary care.

Ease of access to prehabilitation emerged as an important factor. The coronavirus pandemic has accelerated the trend towards remotely delivered interventions, and several of the ongoing trials identified in this review utilise home-based prehabilitation models. Completed studies suggest that home-based multimodal prehabilitation is feasible and leads to improvements in a range of outcomes [51]. However, findings from qualitative studies reveal the importance of accessible support and supervision as a motivator, either through an opportunity to meet others face to face or to monitor and encourage patients to keep on track with their prehabilitation goals. The potential for digital interventions in this field is huge, but lack of access, confidence and competence in relation to technology can present obstacles [31]. Given that gynaecological cancers are more common in those aged 75–79 years old [52, 53], the confidence, skills and access to technology in an older population must be considered.

Whilst there will be emerging evidence from ongoing randomised controlled trials, the heterogeneity of study designs, programme settings, participant eligibility criteria and measured outcomes is significant. The majority of multimodal prehabilitation programmes do incorporate trimodal components encompassing physical activity, nutrition, and psychological interventions. Some also include smoking and alcohol cessation and medication reconciliation whilst others omit the psychological component of prehabilitation. The way in which the individual components of the programme are delivered and what is expected of patients also differ widely across the trials. Outcome measures for post-operative complications, cardiovascular health, functional activity, and health related quality of life are generally included in most studies, however, there are no two trials which have the same set of primary and secondary outcomes.

The lack of standardisation across interventions and outcome measures means that concluding benefit in future work through a meta-analyses may prove challenging. The inability to draw significant improvement benefit of prehabilitation due to the heterogeneity of studies was recently seen in a systematic review in hepatobiliary cancers [54] and has led to a call for standardisation amongst the colorectal community [55]. Greater consistency of outcome measures would also strengthen the evidence base in gynaecological cancer.

It is worth highlighting that the majority of ongoing studies focus on patients with ovarian cancer rather than other gynaecological cancers. This is unsurprising given the high incidence of comorbidity and sarcopenia in this group, as well as the need for pre-operative conditioning prior to major abdominal surgery [7]. However, some cancer centres work under guidance to perform primary debulking surgery for ovarian cancer within two weeks from diagnosis [56], which leaves a very short window of opportunity for prehabilitation. The findings of this review suggest that women may find it difficult to achieve prehabilitation goals as well as to come to terms with diagnosis and prepare for ‘life and death’ during this limited period. Prehabilitation programmes may also need to address issues that are at the forefront of patients’ minds, including socialising, domestic tasks, financial preparation and legal paperwork [39, 41].

Qualitative studies included in this review have focussed primarily on White cohorts [39–43]. Although the incidence of gynaecological cancers is greater amongst White women in the UK [57], there is evidence of increasing incidence and mortality related to endometrial cancer in Black women [57]. This points to a need to ensure that future studies reflect our diverse population and shed light on the factors which influence engagement with prehabilitation amongst different racial groups and ethnic minorities.

## Conclusion

This scoping review illustrates that the evidence for prehabilitation in gynaecological cancer patients is limited, although there are several randomised controlled trials underway. Since a standardised and well accepted prehabilitation programme for this cohort does not yet exist, healthcare organisations and researchers should consider the factors affecting the delivery and engagement of health professionals and patients when designing one. This means taking in to account the needs, knowledge and capacity of healthcare professionals as well as the practical considerations around patient accessibility and acceptability of prehabilitation in the context of wider preparation following a cancer diagnosis. The findings of this review provide important insights into these issues.

## Supplementary Information

**Additional File 1**. Extended Search Strategy. Tables highlighting the detailed search strategy for each repository and the removal of deduplications during screening.

## Data Availability

All data generated or analysed during this study are included in this published article [and its supplementary information files.

## Abbreviations

5STS: Five times sit to stand
6MWT: Six-minute walk test
BMI: Body mass index
CASP: Critical appraisal skills programme
ERAS: Enhanced recovery after surgery
FACT-G: Functional assessment of cancer therapy-general
FRAIL scale: Fatigue, resistance, ambulation, illnesses, and loss of weight
GLIM: Global leadership initiative on malnutrition
HADS: Hospital anxiety and depression scale
IPAQ: International physical activity questionnaire
JBI: Joanna Briggs institute
MUST: Malnutrition universal screening tool
NACT: Neoadjuvant chemotherapy
PG SGA-SF: Patient generated subjective global assessment short form
PRISMA: Preferred reporting items for systematic reviews
QLQ-C30: Quality of life questionnaire for cancer patients
RBANS: Repeatable battery for the assessment of neuropsychological status
RAMSES: Realist and meta-narrative evidence syntheses-evolving standards
SF-12: 12-item short form survey
SF-36: 36-item short form survey
SQUASH: Short questionnaire to assess health enhancing physical activity
TIDieR: Template for intervention description and replication
VO_2_ max: Maximum oxygen consumption
WAIS: Wechsler adult intelligence scale
