# Supplementary material for: Considerations for multimodal prehabilitation in women with gynaecological cancers: a scoping review using realist principles
Source: BMC Womens Health. 2022 Jul 19;22:300. doi: 10.1186/s12905-022-01882-z (PMC9294794; doi:10.1186/s12905-022-01882-z)
Supplement: Supplementary file 1 — Additional File 1. Extended Search Strategy. Tables highlighting the detailed search strategy for each repository and the removal of deduplications during screening. [file 12905_2022_1882_MOESM1_ESM.docx]

| **Medical Databases:** | **Search Strategy** |
| --- | --- |
| MEDLINE, Embase, Emcare, CINAHL, AMED, BNI, PsycINFO | cancer* OR neoplas* OR malignan* OR carcinoma* OR tumo?r* OR oncolog* OR carcinocyte*  (malignant AND tumo?r*) OR (malignant AND neoplasm*)  gyn?ecolog* OR ovar* OR uter* OR womb OR endometr* OR vagin* OR cervi* OR vulva* OR fallopian  prehab OR prehabilitation OR preoperative rehab OR preoperative rehabilitation OR pre-operative rehab OR pre-operative rehabilitation. |
| **Grey Literature Repositories:** |  |
| Cochrane | The original database search was replicated in Cochrane, followed by the addition of filters “gynaecological” and “cancer”. |
| NICE/TRIP | The major search terms did not produce any results. ‘Gynaecology’ was removed from the search to produce a number of results, following which articles making reference in the title to a non-gynaecological cancer were removed. |
| Google | “Prehabilitation AND Gynaecologic Cancer” were used as search terms and relevant links were chosen from 8 pages of search engine results. |

**Supplementary Table 1**: Search strategies used for each repository

**Supplementary Table 2.** Detailed search strategy including the keywords searched in titles and abstracts and subject headings for each repository

| **#** | **Database** | **Search term** | **Results** |
| --- | --- | --- | --- |
| 1 | Medline | (cancer* OR neoplas* OR malignan* OR carcinoma* OR tumo?r* OR oncolog* OR carcinocyte*).ti,ab | 3529311 |
| 2 | Medline | ((malignant AND tumo?r*) OR (malignant AND neoplasm*)).ti,ab | 227631 |
| 3 | Medline | exp NEOPLASMS/ | 3525717 |
| 4 | Medline | (1 OR 2 OR 3) | 4594380 |
| 5 | Medline | (gyn?ecolog* OR ovar* OR uter* OR womb OR endometr* OR vagin* OR cervi* OR vulva* OR fallopian).ti,ab | 866076 |
| 6 | Medline | GYNECOLOGY/ OR "GENITAL DISEASES, FEMALE"/ OR exp UTERUS/ OR exp OVARY/ OR exp VAGINA/ OR exp VULVA/ OR "FALLOPIAN TUBES"/ | 282397 |
| 7 | Medline | (5 OR 6) | 945115 |
| 8 | Medline | (4 AND 7) | 364400 |
| 9 | Medline | (ovar* AND (carcinoma* OR cancer* OR neoplasm* OR tumo?r*)).ti,ab | 113459 |
| 10 | Medline | ((uter* OR womb OR endometr*) AND (carcinoma* OR cancer* OR neoplasm* OR tumo?r*)).ti,ab | 79232 |
| 11 | Medline | (vagin* AND (carcinoma* OR cancer* OR neoplasm* OR tumo?r*)).ti,ab | 15617 |
| 12 | Medline | (cervi* AND (carcinoma* OR cancer* OR neoplasm* OR tumo?r*)).ti,ab | 109173 |
| 13 | Medline | (vulva* AND (carcinoma* OR cancer* OR neoplasm* OR tumo?r*)).ti,ab | 8124 |
| 14 | Medline | (fallopian AND (carcinoma* OR cancer* OR neoplasm* OR tumo?r*)).ti,ab | 3973 |
| 15 | Medline | ((ovar* OR uter* OR womb OR endometr* OR vagin* OR cervi* OR vulva* OR fallopian) ADJ3 (carcinoma* OR cancer* OR neoplasm* OR tumo?r*)).ti,ab | 213879 |
| 16 | Medline | exp "OVARIAN NEOPLASMS"/ OR "FALLOPIAN TUBE NEOPLASMS"/ OR exp "UTERINE NEOPLASMS"/ OR "VAGINAL NEOPLASMS"/ OR "VULVAR NEOPLASMS"/ OR "GENITAL NEOPLASMS, FEMALE"/ | 239275 |
| 17 | Medline | (9 OR 10 OR 11 OR 12 OR 13 OR 14 OR 15 OR 16) | 349927 |
| 18 | Medline | (8 OR 17) | 400460 |
| 19 | Medline | (prehab OR prehabilitation OR preoperative rehab OR preoperative rehabilitation OR pre-operative rehab OR pre-operative rehabilitation).ti,ab | 4389 |
| 20 | Medline | "PREOPERATIVE EXERCISE"/ | 106 |
| 21 | Medline | (preoperative exercise OR pre-operative exercise OR preoperative conditioning OR pre-operative conditioning).ti,ab | 2527 |
| 22 | Medline | (19 OR 20 OR 21) | 6518 |
| 23 | Medline | (18 AND 22) | 55 |
| 24 | Medline | 23 [Languages English] | 45 |
| 25 | EMBASE | (cancer* OR neoplas* OR malignan* OR carcinoma* OR tumo?r* OR oncolog* OR carcinocyte*).ti,ab | 4729710 |
| 26 | EMBASE | ((malignant AND tumo?r*) OR (malignant AND neoplasm*)).ti,ab | 316494 |
| 27 | EMBASE | exp "MALIGNANT NEOPLASM"/ OR "MALIGNANT NEOPLASM, SOLID"/ | 3708917 |
| 28 | EMBASE | (25 OR 26 OR 27) | 5469775 |
| 29 | EMBASE | (gyn?ecolog* OR ovar* OR uter* OR womb OR endometr* OR vagin* OR cervi* OR vulva* OR fallopian).ti,ab | 1139060 |
| 30 | EMBASE | GYNECOLOGY/ OR exp "GYNECOLOGIC DISEASE"/ | 704990 |
| 31 | EMBASE | "GENITAL HIATUS"/ OR "FEMALE GENITAL SYSTEM"/ OR exp "UTERINE ADNEXA"/ OR exp UTERUS/ OR exp VAGINA/ OR exp VULVA/ | 391937 |
| 32 | EMBASE | (29 OR 30 OR 31) | 1477758 |
| 33 | EMBASE | (28 AND 32) | 515857 |
| 34 | EMBASE | (ovar* AND (carcinoma* OR cancer* OR neoplasm* OR tumo?r*)).ti,ab | 165349 |
| 35 | EMBASE | ((uter* OR womb OR endometr*) AND (carcinoma* OR cancer* OR neoplasm* OR tumo?r*)).ti,ab | 108538 |
| 36 | EMBASE | (vagin* AND (carcinoma* OR cancer* OR neoplasm* OR tumo?r*)).ti,ab | 25188 |
| 37 | EMBASE | (cervi* AND (carcinoma* OR cancer* OR neoplasm* OR tumo?r*)).ti,ab | 145603 |
| 38 | EMBASE | (vulva* AND (carcinoma* OR cancer* OR neoplasm* OR tumo?r*)).ti,ab | 11791 |
| 39 | EMBASE | (fallopian AND (carcinoma* OR cancer* OR neoplasm* OR tumo?r*)).ti,ab | 6655 |
| 40 | EMBASE | ((ovar* OR uter* OR womb OR endometr* OR vagin* OR cervi* OR vulva* OR fallopian) ADJ3 (carcinoma* OR cancer* OR neoplasm* OR tumo?r*)).ti,ab | 278664 |
| 41 | EMBASE | "FEMALE GENITAL TRACT CANCER"/ OR "ENDOMETRIOID CARCINOMA"/ OR "FEMALE GENITAL TRACT CARCINOMA"/ OR exp "OVARY CANCER"/ OR exp "UTERUS CANCER"/ OR exp "VAGINA CANCER"/ OR exp "VULVA CANCER"/ OR "UTERINE TUBE CARCINOMA"/ | 290263 |
| 42 | EMBASE | (34 OR 35 OR 36 OR 37 OR 38 OR 39 OR 40 OR 41) | 447378 |
| 43 | EMBASE | (33 OR 42) | 516044 |
| 44 | EMBASE | (prehab OR prehabilitation OR preoperative rehab OR preoperative rehabilitation OR pre-operative rehab OR pre-operative rehabilitation).ti,ab | 1435 |
| 45 | EMBASE | (preoperative exercise OR pre-operative exercise OR preoperative conditioning OR pre-operative conditioning).ti,ab | 476 |
| 46 | EMBASE | "PREOPERATIVE EXERCISE"/ | 249 |
| 47 | EMBASE | (44 OR 45 OR 46) | 1855 |
| 48 | EMBASE | (43 AND 47) | 35 |
| 49 | EMBASE | 48 [Languages English] | 34 |
| 50 | EMCARE | (cancer* OR neoplas* OR malignan* OR carcinoma* OR tumo?r* OR oncolog* OR carcinocyte*).ti,ab | 714885 |
| 51 | EMCARE | ((malignant AND tumo?r*) OR (malignant AND neoplasm*)).ti,ab | 39211 |
| 52 | EMCARE | exp "MALIGNANT NEOPLASM"/ OR "MALIGNANT NEOPLASM, SOLID"/ | 587814 |
| 53 | EMCARE | (50 OR 51 OR 52) | 833263 |
| 54 | EMCARE | (gyn?ecolog* OR ovar* OR uter* OR womb OR endometr* OR vagin* OR cervi* OR vulva* OR fallopian).ti,ab | 195638 |
| 55 | EMCARE | GYNECOLOGY/ OR exp "GYNECOLOGIC DISEASE"/ | 138634 |
| 56 | EMCARE | "FEMALE GENITAL SYSTEM"/ OR "GENITAL HIATUS"/ OR exp UTERUS/ OR exp VAGINA/ OR exp VULVA/ OR exp "UTERINE ADNEXA"/ | 39957 |
| 57 | EMCARE | (54 OR 55 OR 56) | 253570 |
| 67 | EMCARE | (53 AND 57) | 84177 |
| 58 | EMCARE | (ovar* AND (carcinoma* OR cancer* OR neoplasm* OR tumo?r*)).ti,ab | 21075 |
| 59 | EMCARE | ((uter* OR womb OR endometr*) AND (carcinoma* OR cancer* OR neoplasm* OR tumo?r*)).ti,ab | 14229 |
| 60 | EMCARE | (vagin* AND (carcinoma* OR cancer* OR neoplasm* OR tumo?r*)).ti,ab | 3826 |
| 61 | EMCARE | (cervi* AND (carcinoma* OR cancer* OR neoplasm* OR tumo?r*)).ti,ab | 25179 |
| 62 | EMCARE | (vulva* AND (carcinoma* OR cancer* OR neoplasm* OR tumo?r*)).ti,ab | 1479 |
| 63 | EMCARE | (fallopian AND (carcinoma* OR cancer* OR neoplasm* OR tumo?r*)).ti,ab | 829 |
| 64 | EMCARE | ((ovar* OR uter* OR womb OR endometr* OR vagin* OR cervi* OR vulva* OR fallopian) ADJ3 (carcinoma* OR cancer* OR neoplasm* OR tumo?r*)).ti,ab | 41837 |
| 65 | EMCARE | "FEMALE GENITAL TRACT CANCER"/ OR "ENDOMETRIOID CARCINOMA"/ OR "FEMALE GENITAL TRACT CARCINOMA"/ OR exp "OVARY CANCER"/ OR exp "UTERUS CANCER"/ OR exp "VAGINA CANCER"/ OR exp "VULVA CANCER"/ OR "UTERINE TUBE CARCINOMA"/ | 52983 |
| 66 | EMCARE | (58 OR 59 OR 60 OR 61 OR 62 OR 63 OR 64 OR 65) | 71635 |
| 68 | EMCARE | (67 OR 66) | 84219 |
| 69 | EMCARE | (prehab OR prehabilitation OR preoperative rehab OR preoperative rehabilitation OR pre-operative rehab OR pre-operative rehabilitation).ti,ab | 472 |
| 70 | EMCARE | (preoperative exercise OR pre-operative exercise OR preoperative conditioning OR pre-operative conditioning).ti,ab | 128 |
| 71 | EMCARE | "PREOPERATIVE EXERCISE"/ | 39 |
| 72 | EMCARE | (69 OR 70 OR 71) | 580 |
| 73 | EMCARE | (68 AND 72) | 7 |
| 74 | EMCARE | 73 [English language] [Languages English] | 6 |
| 75 | CINAHL | (cancer* OR neoplas* OR malignan* OR carcinoma* OR tumo?r* OR oncolog* OR carcinocyte*).ti,ab | 624103 |
| 76 | CINAHL | ((malignant AND tumo?r*) OR (malignant AND neoplasm*)).ti,ab | 20913 |
| 77 | CINAHL | exp NEOPLASMS/ | 589429 |
| 78 | CINAHL | (75 OR 76 OR 77) | 798250 |
| 79 | CINAHL | (gyn?ecolog* OR ovar* OR uter* OR womb OR endometr* OR vagin* OR cervi* OR vulva* OR fallopian).ti,ab | 162906 |
| 80 | CINAHL | GYNECOLOGY/ OR exp "GENITALIA, FEMALE"/ | 27169 |
| 81 | CINAHL | "GENITAL DISEASES, FEMALE"/ OR "UTERINE DISEASES"/ OR "VAGINAL DISEASES"/ OR "VULVAR DISEASES"/ OR exp "ADNEXAL DISEASES"/ | 28535 |
| 82 | CINAHL | (79 OR 80 OR 81) | 176444 |
| 83 | CINAHL | (78 AND 82) | 67143 |
| 84 | CINAHL | (ovar* AND (carcinoma* OR cancer* OR neoplasm* OR tumo?r*)).ti,ab | 17321 |
| 85 | CINAHL | ((uter* OR womb OR endometr*) AND (carcinoma* OR cancer* OR neoplasm* OR tumo?r*)).ti,ab | 11192 |
| 86 | CINAHL | (vagin* AND (carcinoma* OR cancer* OR neoplasm* OR tumo?r*)).ti,ab | 2656 |
| 87 | CINAHL | (cervi* AND (carcinoma* OR cancer* OR neoplasm* OR tumo?r*)).ti,ab | 20418 |
| 88 | CINAHL | (vulva* AND (carcinoma* OR cancer* OR neoplasm* OR tumo?r*)).ti,ab | 1247 |
| 89 | CINAHL | (fallopian AND (carcinoma* OR cancer* OR neoplasm* OR tumo?r*)).ti,ab | 651 |
| 90 | CINAHL | ((ovar* OR uter* OR womb OR endometr* OR vagin* OR cervi* OR vulva* OR fallopian) ADJ3 (carcinoma* OR cancer* OR neoplasm* OR tumo?r*)).ti,ab | 37586 |
| 91 | CINAHL | "GENITAL NEOPLASMS, FEMALE"/ OR exp "OVARIAN NEOPLASMS"/ OR "UTERINE NEOPLASMS"/ OR exp "CERVIX NEOPLASMS"/ OR "ENDOMETRIAL NEOPLASMS"/ OR "VAGINAL NEOPLASMS"/ OR "VULVAR NEOPLASMS"/ | 41928 |
| 92 | CINAHL | (84 OR 85 OR 86 OR 87 OR 88 OR 89 OR 90 OR 91) | 60154 |
| 93 | CINAHL | (83 OR 92) | 72193 |
| 94 | CINAHL | (prehab OR prehabilitation OR preoperative rehab OR preoperative rehabilitation OR pre-operative rehab OR pre-operative rehabilitation).ti,ab | 1646 |
| 95 | CINAHL | (preoperative exercise OR pre-operative exercise OR preoperative conditioning OR pre-operative conditioning).ti,ab | 856 |
| 96 | CINAHL | PREHABILITATION/ | 171 |
| 97 | CINAHL | (94 OR 95 OR 96) | 2345 |
| 98 | CINAHL | (93 AND 97) | 16 |
| 99 | CINAHL | 98 [Languages eng] | 15 |
| 100 | AMED | (cancer* OR neoplas* OR malignan* OR carcinoma* OR tumo?r* OR oncolog* OR carcinocyte*).ti,ab | 19421 |
| 101 | AMED | ((malignant AND tumo?r*) OR (malignant AND neoplasm*)).ti,ab | 438 |
| 102 | AMED | exp NEOPLASMS/ | 16752 |
| 103 | AMED | (100 OR 101 OR 102) | 22796 |
| 104 | AMED | (gyn?ecolog* OR ovar* OR uter* OR womb OR endometr* OR vagin* OR cervi* OR vulva* OR fallopian).ti,ab | 5929 |
| 105 | AMED | "GENITAL DISEASES FEMALE"/ OR "OVARIAN DISEASE"/ OR "UTERINE DISEASE"/ OR "UTERINE CERVICAL DISEASE"/ OR "VAGINAL DISEASE"/ OR VAGINITIS/ | 580 |
| 106 | AMED | (104 OR 105) | 6193 |
| 107 | AMED | (103 AND 106) | 918 |
| 108 | AMED | (ovar* AND (carcinoma* OR cancer* OR neoplasm* OR tumo?r*)).ti,ab | 301 |
| 109 | AMED | ((uter* OR womb OR endometr*) AND (carcinoma* OR cancer* OR neoplasm* OR tumo?r*)).ti,ab | 114 |
| 110 | AMED | (vagin* AND (carcinoma* OR cancer* OR neoplasm* OR tumo?r*)).ti,ab | 18 |
| 111 | AMED | (cervi* AND (carcinoma* OR cancer* OR neoplasm* OR tumo?r*)).ti,ab | 310 |
| 112 | AMED | (vulva* AND (carcinoma* OR cancer* OR neoplasm* OR tumo?r*)).ti,ab | 10 |
| 113 | AMED | (fallopian AND (carcinoma* OR cancer* OR neoplasm* OR tumo?r*)).ti,ab | 1 |
| 114 | AMED | ((ovar* OR uter* OR womb OR endometr* OR vagin* OR cervi* OR vulva* OR fallopian) ADJ3 (carcinoma* OR cancer* OR neoplasm* OR tumo?r*)).ti,ab | 516 |
| 115 | AMED | "GENITAL NEOPLASMS FEMALE"/ OR "OVARIAN NEOPLASMS"/ OR "UTERINE NEOPLASMS"/ OR "UTERINE CERVICAL NEOPLASMS"/ | 341 |
| 116 | AMED | (108 OR 109 OR 110 OR 111 OR 112 OR 113 OR 114 OR 115) | 777 |
| 117 | AMED | (107 OR 116) | 946 |
| 118 | AMED | (prehab OR prehabilitation OR preoperative rehab OR preoperative rehabilitation OR pre-operative rehab OR pre-operative rehabilitation).ti,ab | 45 |
| 119 | AMED | (preoperative exercise OR pre-operative exercise OR preoperative conditioning OR pre-operative conditioning).ti,ab | 13 |
| 120 | AMED | "PREOPERATIVE CARE"/ | 357 |
| 121 | AMED | (118 OR 119 OR 120) | 392 |
| 122 | AMED | (117 AND 121) | 2 |
| 123 | BNI | (cancer* OR neoplas* OR malignan* OR carcinoma* OR tumo?r* OR oncolog* OR carcinocyte*).ti,ab | 66917 |
| 124 | BNI | ((malignant AND tumo?r*) OR (malignant AND neoplasm*)).ti,ab | 685 |
| 125 | BNI | CANCER/ | 28654 |
| 126 | BNI | (123 OR 124 OR 125) | 70235 |
| 127 | BNI | (gyn?ecolog* OR ovar* OR uter* OR womb OR endometr* OR vagin* OR cervi* OR vulva* OR fallopian).ti,ab | 17510 |
| 128 | BNI | GYNECOLOGY/ | 3417 |
| 129 | BNI | UTERUS/ OR ENDOMETRIUM/ OR CERVIX/ OR "FALLOPIAN TUBES"/ OR OVARIES/ OR VAGINA/ | 1569 |
| 130 | BNI | (127 OR 128 OR 129) | 19156 |
| 131 | BNI | (126 AND 130) | 5986 |
| 132 | BNI | (ovar* AND (carcinoma* OR cancer* OR neoplasm* OR tumo?r*)).ti,ab | 1730 |
| 133 | BNI | ((uter* OR womb OR endometr*) AND (carcinoma* OR cancer* OR neoplasm* OR tumo?r*)).ti,ab | 777 |
| 134 | BNI | (vagin* AND (carcinoma* OR cancer* OR neoplasm* OR tumo?r*)).ti,ab | 291 |
| 135 | BNI | (cervi* AND (carcinoma* OR cancer* OR neoplasm* OR tumo?r*)).ti,ab | 2597 |
| 136 | BNI | (vulva* AND (carcinoma* OR cancer* OR neoplasm* OR tumo?r*)).ti,ab | 125 |
| 137 | BNI | (fallopian AND (carcinoma* OR cancer* OR neoplasm* OR tumo?r*)).ti,ab | 31 |
| 138 | BNI | ((ovar* OR uter* OR womb OR endometr* OR vagin* OR cervi* OR vulva* OR fallopian) ADJ3 (carcinoma* OR cancer* OR neoplasm* OR tumo?r*)).ti,ab | 4402 |
| 139 | BNI | "OVARIAN CANCER"/ OR "CERVICAL CANCER"/ OR "ENDOMETRIAL CANCER"/ OR "UTERINE CANCER"/ OR "GENITAL CANCERS"/ | 4290 |
| 140 | BNI | (132 OR 133 OR 134 OR 135 OR 136 OR 137 OR 138 OR 139) | 6411 |
| 141 | BNI | (131 OR 140) | 7300 |
| 142 | BNI | (prehab OR prehabilitation OR preoperative rehab OR preoperative rehabilitation OR pre-operative rehab OR pre-operative rehabilitation).ti,ab | 94 |
| 143 | BNI | (preoperative exercise OR pre-operative exercise OR preoperative conditioning OR pre-operative conditioning).ti,ab | 69 |
| 144 | BNI | REHABILITATION/ | 8898 |
| 145 | BNI | (142 OR 143 OR 144) | 9020 |
| 146 | BNI | (141 AND 145) | 32 |
| 147 | PsycINFO | (cancer* OR neoplas* OR malignan* OR carcinoma* OR tumo?r* OR oncolog* OR carcinocyte*).ti,ab | 86012 |
| 148 | PsycINFO | ((malignant AND tumo?r*) OR (malignant AND neoplasm*)).ti,ab | 1430 |
| 149 | PsycINFO | exp NEOPLASMS/ | 55351 |
| 150 | PsycINFO | (147 OR 148 OR 149) | 89475 |
| 151 | PsycINFO | (gyn?ecolog* OR ovar* OR uter* OR womb OR endometr* OR vagin* OR cervi* OR vulva* OR fallopian).ti,ab | 29002 |
| 152 | PsycINFO | GYNECOLOGY/ | 1170 |
| 153 | PsycINFO | "FEMALE GENITALIA"/ OR OVARIES/ OR UTERUS/ OR CERVIX/ OR VAGINA/ OR exp "GYNECOLOGICAL DISORDERS"/ | 6612 |
| 154 | PsycINFO | (151 OR 152 OR 153) | 31359 |
| 155 | PsycINFO | (150 AND 154) | 5749 |
| 156 | PsycINFO | (ovar* AND (carcinoma* OR cancer* OR neoplasm* OR tumo?r*)).ti,ab | 1354 |
| 157 | PsycINFO | ((uter* OR womb OR endometr*) AND (carcinoma* OR cancer* OR neoplasm* OR tumo?r*)).ti,ab | 584 |
| 158 | PsycINFO | (vagin* AND (carcinoma* OR cancer* OR neoplasm* OR tumo?r*)).ti,ab | 251 |
| 159 | PsycINFO | (cervi* AND (carcinoma* OR cancer* OR neoplasm* OR tumo?r*)).ti,ab | 3001 |
| 160 | PsycINFO | (vulva* AND (carcinoma* OR cancer* OR neoplasm* OR tumo?r*)).ti,ab | 82 |
| 161 | PsycINFO | (fallopian AND (carcinoma* OR cancer* OR neoplasm* OR tumo?r*)).ti,ab | 12 |
| 162 | PsycINFO | ((ovar* OR uter* OR womb OR endometr* OR vagin* OR cervi* OR vulva* OR fallopian) ADJ3 (carcinoma* OR cancer* OR neoplasm* OR tumo?r*)).ti,ab | 4060 |
| 163 | PsycINFO | (156 OR 157 OR 158 OR 159 OR 160 OR 161 OR 162) | 4722 |
| 164 | PsycINFO | (155 OR 163) | 5749 |
| 165 | PsycINFO | (prehab OR prehabilitation OR preoperative rehab OR preoperative rehabilitation OR pre-operative rehab OR pre-operative rehabilitation).ti,ab | 167 |
| 166 | PsycINFO | (preoperative exercise OR pre-operative exercise OR preoperative conditioning OR pre-operative conditioning).ti,ab | 67 |
| 167 | PsycINFO | exp EXERCISE/ OR REHABILITATION/ | 49573 |
| 168 | PsycINFO | "PREOPERATIVE PERIOD"/ OR exp "PHYSICAL ACTIVITY"/ | 46280 |
| 169 | PsycINFO | (165 OR 166 OR 167 OR 168) | 67336 |
| 170 | PsycINFO | (164 AND 169) | 94 |
| 171 | PsycINFO | 170 [Languages English] | 93 |
| 172 | Medline | (18 AND 19) | 43 |
| 173 | Medline | 172 [Languages English] | 39 |
| 174 | EMBASE | (43 AND 44) | 33 |
| 175 | EMBASE | 174 [English language] [Languages English] | 32 |
| 176 | EMCARE | (68 AND 69) | 7 |
| 177 | EMCARE | 176 [English language] [Languages English] | 6 |
| 178 | CINAHL | (93 AND 94) | 13 |
| 179 | CINAHL | 178 [Languages eng] | 13 |
| 180 | AMED | (117 AND 118) | 0 |
| 181 | BNI | (141 AND 142) | 1 |
| 182 | PsycINFO | (164 AND 165) | 5 |

The search was started following an initial discussion with the user over how to use the search resource and how to structure a search strategy, which included an initial discussion on what search terms might be relevant to use. Following a second discussion, it was agreed that the term "prehab" and variations thereof was the most relevant to use, as more specific terms caused a large number of irrelevant results to come out. Once a basic outline was agreed upon, the search strategy was devised taking three major elements as search terms:

1. "Cancer AND Gynaecology"

2. "Gynaecological Cancers"

3. "Prehabilitation"

The first two were searched for individually, and then combined using OR, with the total results then combined with Prehabilitation. This can be seen in greater detail in the finalised search strategy. This was initially worked out, and approved by the user following discussion with the limits of "English Language", "female" and "adult" added. However, after an initial test, it was determined that including these last two as limits in the search itself removed too many potentially relevant results, and so were removed from the final search strategy. The search was then run in a total of seven databases using the HDAS platform:

• MEDLINE

• Embase

• Emcare

• CINAHL

• AMED

• BNI

• PsycINFO

The overall search produced a total of 227 results from the seven databases. Of these, 48 were duplicates and were removed using HDAS's deduplication function, to leave a final total of 179 results. These have been presented within this document, and have also been included in both Excel format, to allow for ease of manipulation, and RIS format for use in a reference management package.

Following the receipt of the results after the original search, the user subsequently requested a slight revision to the end of the strategy, to remove reference to pre-operative exercise specifically, which had been included as an area related to prehabilitation, and only use the general term “prehabilitation” and variations thereof. This produced a total of 97 results across the seven databases, with 23 duplicates removed using HDAS’s deduplication function, to leave 74 results.

Each database was searched individually, both using keywords and the database’s own controlled vocabulary – the HDAS platform allowed results from multiple databases to be collated together into a single list, which could then be collated together into a single list of results. This list could then be deduplicated using HDAS’s own deduplication function.

Since the search was conducted, HDAS has been discontinued, however, the underlying databases remain available via other platforms such as Ovid, EBSCO and ProQuest and the same search strategy can be replicated.

The search was also conducted in the Cochrane Library platform, across the Cochrane Database of Systematic Reviews, Central Register of Clinical Trials and Cochrane Clinical Answers, using an identical set of keywords and subject headings to the MEDLINE version of the original search. Additionally a search for grey literature was conducted in NICE Evidence Search and TRIP Database, and using the search engine Google. The results obtained from these sources were combined with the results from the bibliographic database search in Zotero, a reference management software, and then subjected to further deduplication.

**Supplementary Table 3.** Repositories searched and numbers of articles retrieved following deduplication

|  | **Results from initial search** | **Results after deduplication** |
| --- | --- | --- |
| MEDLINE | 39 | 37 |
| Embase | 32 | 18 |
| Emcare | 6 | 0 |
| CINAHL | 13 | 2 |
| AMED | 0 | 0 |
| BNI | 1 | 0 |
| PsycINFO | 5 | 2 |
| Cochrane | 20 | 20 |
| Google | 14 | 11 |
| NICE | 13 | 13 |
| TRIP | 6 | 6 |

**SupplementaryTable 4.** Preferred Reporting Items for Systematic reviews and Meta-Analyses extension for Scoping Reviews (PRISMA-ScR) Checklist

| **SECTION** | **ITEM** | **PRISMA-ScR CHECKLIST ITEM** | **REPORTED ON PAGE #** |
| --- | --- | --- | --- |
| **TITLE** | | | |
| Title | 1 | Identify the report as a scoping review. | 1 |
| **ABSTRACT** | | | |
| Structured summary | 2 | Provide a structured summary that includes (as applicable): background, objectives, eligibility criteria, sources of evidence, charting methods, results, and conclusions that relate to the review questions and objectives. | 2-3 |
| **INTRODUCTION** | | | |
| Rationale | 3 | Describe the rationale for the review in the context of what is already known. Explain why the review questions/objectives lend themselves to a scoping review approach. | 3-4 |
| Objectives | 4 | Provide an explicit statement of the questions and objectives being addressed with reference to their key elements (e.g., population or participants, concepts, and context) or other relevant key elements used to conceptualize the review questions and/or objectives. | 4 |
| **METHODS** | | | |
| Protocol and registration | 5 | Indicate whether a review protocol exists; state if and where it can be accessed (e.g., a Web address); and if available, provide registration information, including the registration number. | 4-5 |
| Eligibility criteria | 6 | Specify characteristics of the sources of evidence used as eligibility criteria (e.g., years considered, language, and publication status), and provide a rationale. | 6  Supplementary material |
| Information sources* | 7 | Describe all information sources in the search (e.g., databases with dates of coverage and contact with authors to identify additional sources), as well as the date the most recent search was executed. | 7 |
| Search | 8 | Present the full electronic search strategy for at least 1 database, including any limits used, such that it could be repeated. | Supplementary material |
| Selection of sources of evidence† | 9 | State the process for selecting sources of evidence (i.e., screening and eligibility) included in the scoping review. | 7 |
| Data charting process‡ | 10 | Describe the methods of charting data from the included sources of evidence (e.g., calibrated forms or forms that have been tested by the team before their use, and whether data charting was done independently or in duplicate) and any processes for obtaining and confirming data from investigators. | 8 |
| Data items | 11 | List and define all variables for which data were sought and any assumptions and simplifications made. | 9-10 |
| Critical appraisal of individual sources of evidence§ | 12 | If done, provide a rationale for conducting a critical appraisal of included sources of evidence; describe the methods used and how this information was used in any data synthesis (if appropriate). | Not done based on JBI guidelines for scoping review |
| Synthesis of results | 13 | Describe the methods of handling and summarizing the data that were charted. | 9 |
| **RESULTS** | | | |
| Selection of sources of evidence | 14 | Give numbers of sources of evidence screened, assessed for eligibility, and included in the review, with reasons for exclusions at each stage, ideally using a flow diagram. | 9 |
| Characteristics of sources of evidence | 15 | For each source of evidence, present characteristics for which data were charted and provide the citations. | Summary table in supplementary material |
| Critical appraisal within sources of evidence | 16 | If done, present data on critical appraisal of included sources of evidence (see item 12). | Not done although strengths/limitations were provided in above table |
| Results of individual sources of evidence | 17 | For each included source of evidence, present the relevant data that were charted that relate to the review questions and objectives. | Table 2 and 3 |
| Synthesis of results | 18 | Summarize and/or present the charting results as they relate to the review questions and objectives. | 9-20 |
| **DISCUSSION** | | | |
| Summary of evidence | 19 | Summarize the main results (including an overview of concepts, themes, and types of evidence available), link to the review questions and objectives, and consider the relevance to key groups. | 20-24 |
| Limitations | 20 | Discuss the limitations of the scoping review process. | 21 |
| Conclusions | 21 | Provide a general interpretation of the results with respect to the review questions and objectives, as well as potential implications and/or next steps. | 24 |
| **FUNDING** | | | |
| Funding | 22 | Describe sources of funding for the included sources of evidence, as well as sources of funding for the scoping review. Describe the role of the funders of the scoping review. | 25 |

JBI = Joanna Briggs Institute; PRISMA-ScR = Preferred Reporting Items for Systematic reviews and Meta-Analyses extension for Scoping Reviews.

* Where *sources of evidence* (see second footnote) are compiled from, such as bibliographic databases, social media platforms, and Web sites.

† A more inclusive/heterogeneous term used to account for the different types of evidence or data sources (e.g., quantitative and/or qualitative research, expert opinion, and policy documents) that may be eligible in a scoping review as opposed to only studies. This is not to be confused with *information sources* (see first footnote).

‡ The frameworks by Arksey and O’Malley (6) and Levac and colleagues (7) and the JBI guidance (4, 5) refer to the process of data extraction in a scoping review as data charting*.*

§ The process of systematically examining research evidence to assess its validity, results, and relevance before using it to inform a decision. This term is used for items 12 and 19 instead of "risk of bias" (which is more applicable to systematic reviews of interventions) to include and acknowledge the various sources of evidence that may be used in a scoping review (e.g., quantitative and/or qualitative research, expert opinion, and policy document).

*From:* Tricco AC, Lillie E, Zarin W, O'Brien KK, Colquhoun H, Levac D, et al. PRISMA Extension for Scoping Reviews (PRISMAScR): Checklist and Explanation. Ann Intern Med. 2018;169:467–473. [doi: 10.7326/M18-0850](http://annals.org/aim/fullarticle/2700389/prisma-extension-scoping-reviews-prisma-scr-checklist-explanation).
